# Covalent Inhibition of SHMT2 by Gambogic Acid Induces Ferroptosis Through Mitochondrial Collapse in Triple‐Negative Breast Cancer

**DOI:** 10.1002/advs.202520252

**Published:** 2026-06-30

**Authors:** Tong Yang, Chong Qiu, Yulei Li, Ying Zhang, Chen Wang, Jie Zhou, Zheng Chu, Ang Ma, Ling Huang, Yin Kwan Wong, Junzhe Zhang, Peng Gao, Cui Liu, Junhua Zhang, Huan Tang, Jigang Wang

**Affiliations:** ^1^ School of Chinese Materia Medica Tianjin University of Traditional Chinese Medicine Tianjin China; ^2^ State Key Laboratory for Quality Ensurance and Sustainable Use of Dao‐di Herbs, Artemisinin Research Center, and Institute of Chinese Materia Medica China Academy of Chinese Medical Sciences Beijing China; ^3^ Blood Transfusion Department, First Affiliated Hospital of Gannan Medical University, Key Laboratory of Prevention and Treatment of Cardiovascular and Cerebrovascular Diseases, Ministry of Education Gannan Medical University Ganzhou Jiangxi China; ^4^ Department of Pulmonary and Critical Care Medicine, Shenzhen Institute of Respiratory Diseases, Guangdong Provincial Clinical Research Center for Geriatrics, Shenzhen Clinical Research Center for Geriatrics, Shenzhen People's Hospital, The Second Clinical Medical College Jinan University Shenzhen China; ^5^ State Key Laboratory of Chinese Medicine Modernization Tianjin University of Traditional Chinese Medicine Tianjin China; ^6^ Haihe Laboratory of Modern Chinese Medicine Tianjin China

**Keywords:** chemoproteomics, ferroptosis, gambogic acid, mitochondrial metabolism, target identification, triple‐negative breast cancer

## Abstract

Triple‐negative breast cancer (TNBC) is an aggressive subtype lacking targeted therapies due to the absence of hormone receptors and HER2 expression, resulting in poor clinical outcomes and limited treatment options. Identifying novel vulnerabilities is therefore critical to advancing TNBC therapeutics. Mitochondrial metabolism has emerged as a key regulator of cancer cell survival and proliferation, with serine hydroxymethyltransferase 2 (SHMT2) playing a central role in mitochondrial one‐carbon metabolism by supplying one‐carbon units for nucleotide biosynthesis and maintaining redox homeostasis. Despite its established importance in cancer metabolism, the functional role and therapeutic potential of SHMT2 in TNBC remain underexplored. Here, we demonstrate that gambogic acid (GA), a natural product with reported anticancer properties, exerts potent and selective cytotoxicity against TNBC cells by covalently targeting SHMT2. GA binds specifically to the critical cysteine residue Cys241, inhibiting SHMT2 enzymatic activity and disrupting mitochondrial function. This leads to bioenergetic collapse, activation of the Nrf2/HO‐1 axis, iron overload, and induction of ferroptosis, a non‐apoptotic form of cell death increasingly recognized for its therapeutic potential. Our integrative chemoproteomic and mechanistic studies reveal a novel SHMT2‐mitochondria‐Nrf2/HO‐1‐ferroptosis axis driving GA's anti‐TNBC activity. Moreover, SHMT2 overexpression in TNBC correlates with tumor aggressiveness and poor prognosis, underscoring its role as a metabolic oncogene and promising drug target. These findings establish GA as a novel covalent SHMT2 inhibitor and provide a new framework for exploiting metabolic vulnerabilities to overcome TNBC treatment resistance.

## Graphical abstract

In this study, chemoproteomics combined with genetic and functional analyses was integrated to identify SHMT2 as a covalent and functional target of gambogic acid (GA) in triple‐negative breast cancer (TNBC). Further validation demonstrated that GA selectively modifies the Cys241 site of SHMT2, triggering mitochondrial dysfunction, activating the Nrf2/HO‐1 axis, and inducing iron‐dependent ferroptosis, thereby suppressing tumor growth in vitro and in vivo. These findings not only deepen our mechanistic understanding of metabolic vulnerabilities in TNBC but also establish a framework supporting SHMT2‐targeted ferroptosis therapy, providing theoretical groundwork for its clinical application.

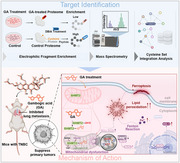

## 1 Introduction

Triple‐negative breast cancer (TNBC) is defined by the lack of estrogen receptor (ER), progesterone receptor (PR), and HER2 expression, and represents an aggressive and clinically challenging breast cancer subtype [1]. Comprising roughly 15–20% of all breast cancer instances, TNBC is linked to a notably unfavorable prognosis, marked by early relapse, a high propensity for metastasis, and the shortest overall survival among breast cancer subtypes [2]. This unfavorable clinical trajectory is primarily attributable to the absence of targetable molecular drivers, which preclude the use of established endocrine and HER2‐targeted therapies effective in other subtypes. As a result, chemotherapy that targets the entire system continues to be the primary approach for treating TNBC. Nevertheless, its effectiveness can frequently be undermined by inherent or developed resistance, considerable toxicity, and elevated occurrences of distant recurrence [3]. For individuals suffering from advanced or metastatic TNBC, the outlook is especially bleak, with median overall survival typically less than 24 months and only marginal improvements achieved over the past decade [4]. This persistent therapeutic gap highlights an urgent and pressing need for innovative, potent, and targeted treatment approaches that take advantage of the unique molecular weaknesses associated with TNBC.

Ferroptosis, which is characterized by the accumulation of harmful lipid peroxides and is regulated in an iron‐dependent manner, has been recognized as a potentially effective treatment strategy for various cancers, such as TNBC [5]. Mechanistically distinct from apoptosis, ferroptosis is governed by the cellular systems that manage antioxidants (such as the glutathione (GSH)/GPX4 pathway) and iron metabolism [5]. Increasing evidence highlights ferroptosis as an intrinsic tumor suppressive mechanism that cooperates with multiple tumor suppressors [6, 7]. Notably, TNBC exhibits distinct molecular signatures associated with increased ferroptosis sensitivity [8, 9], indicating that deliberate induction of ferroptosis may represent a viable approach to overcome the therapy resistance commonly observed in this subtype.

Mitochondria, as central regulators of cellular metabolism and redox homeostasis, play crucial and multifaceted roles in both TNBC pathogenesis and the execution of ferroptosis [10, 11, 12]. In TNBC, dysregulation of mitochondrial metabolic enzymes and dynamics contributes to metabolic reprogramming, supporting tumor growth and aggressive phenotypes [13, 14, 15]. Mitochondrial dysfunction, in particular, serves as a potent trigger for ferroptosis by disrupting iron homeostasis, increasing reactive oxygen species (ROS) production, and impairing antioxidant defenses [16, 17]. Thus, therapeutically targeting key mitochondrial metabolic enzymes to induce dysfunction and trigger ferroptosis holds substantial promise for TNBC treatment. Serine hydroxymethyltransferase 2 (SHMT2), a pivotal enzyme in mitochondrial one‐carbon metabolism, exemplifies such a target. SHMT2 supports nucleotide biosynthesis and redox balance, thereby sustaining critical pathways required for cancer cell survival and proliferation [18, 19]. SHMT2 is often amplified in breast cancer and correlates with poor prognosis, especially in ER‐negative subtypes [20, 21]. Its inhibition has been linked to mitochondrial dysfunction and ROS accumulation, hallmarks of ferroptosis induction [22, 23, 24]. However, a direct causal relationship between SHMT2 inhibition and ferroptosis in TNBC has not been conclusively demonstrated, and potent, selective SHMT2 inhibitors capable of leveraging this vulnerability remain lacking. Addressing this gap is essential for the development of mitochondria‐targeted, ferroptosis‐inducing therapies for TNBC.

Gambogic acid (GA) is a naturally occurring polyprenylated xanthone that is derived from the resin of *Garcinia hanburyi*, demonstrates wide‐ranging antitumor effects against diverse cancer types, such as those of the lung, prostate, liver, and hematopoietic system [25, 26, 27, 28, 29]. This potent bioactivity has prompted extensive investigation into its molecular mechanisms of action. Several protein targets of GA have been identified, including heat‐shock protein 90 (Hsp90) [30], ubiquitin‐specific protease 2 (USP2) [31], and 6‐phosphogluconate dehydrogenase (6PGD) [27], implicating GA in the disruption of protein folding, ubiquitin signaling, and pentose phosphate pathway metabolism, respectively. Notably, GA contains an α, β‐unsaturated carbonyl moiety that enables it to act as an electrophilic Michael acceptor, covalently modifying nucleophilic cysteine residues on target proteins, a mechanism increasingly recognized as a powerful approach for designing potent and selective inhibitors [32]. Despite these advances, the therapeutic potential of GA in TNBC, including its specific molecular targets and mechanisms of action within this aggressive subtype, remains largely unexplored. Although GA exhibits cytotoxicity in various cancer models, its activity against TNBC has not been systematically characterized, and its covalent target landscape in this context remains undefined. Critically, the potential for GA's reactive pharmacophore to engage novel, context‐specific targets in TNBC, particularly those related to mitochondrial vulnerabilities or ferroptosis, has yet to be fully leveraged.

Here, we demonstrate that GA effectively suppresses the progression of TNBC by covalently targeting SHMT2, a key mitochondrial metabolic enzyme, thereby inducing mitochondrial dysfunction and triggering ferroptosis. Using a reactive cysteine‐directed chemoproteomic approach, we identified SHMT2 as the primary covalent target of GA in TNBC cells. Mechanistic validation confirmed that GA covalently modifies SHMT2 at Cys241, thereby suppressing both its enzymatic function and its ability to form oligomers. Inhibition of SHMT2 disrupts mitochondrial one‐carbon metabolism, leading to a collapse in bioenergetics, impaired antioxidant defenses, and mitochondrial structural disintegration. This mitochondrial failure activates the Nrf2/HO‐1 stress response pathway, which paradoxically promotes heme degradation and iron release. The resulting iron overload, combined with compromised antioxidant capacity, drives excessive lipid peroxidation, culminating in ferroptosis. Importantly, we validated this mechanism in vivo, demonstrating that GA significantly inhibits orthotopic TNBC tumor growth and pulmonary metastasis. Additionally, we identified SHMT2 overexpression as a defining feature of TNBC, correlating with poor clinical prognosis, and confirmed its essential oncogenic role in maintaining mitochondrial integrity, preventing ferroptosis, and supporting tumor progression. Collectively, these findings uncover a novel mitochondria‐targeted, ferroptosis‐inducing mechanism of GA, provide a compelling rationale for its development as a therapeutic agent in TNBC, and establish the SHMT2‐ferroptosis axis as a fundamental and actionable vulnerability in this treatment‐refractory cancer.

## 2 Results

### 2.1 Gambogic Acid Effectively Suppresses TNBC Cell Proliferation In Vitro

**FIGURE 1:**
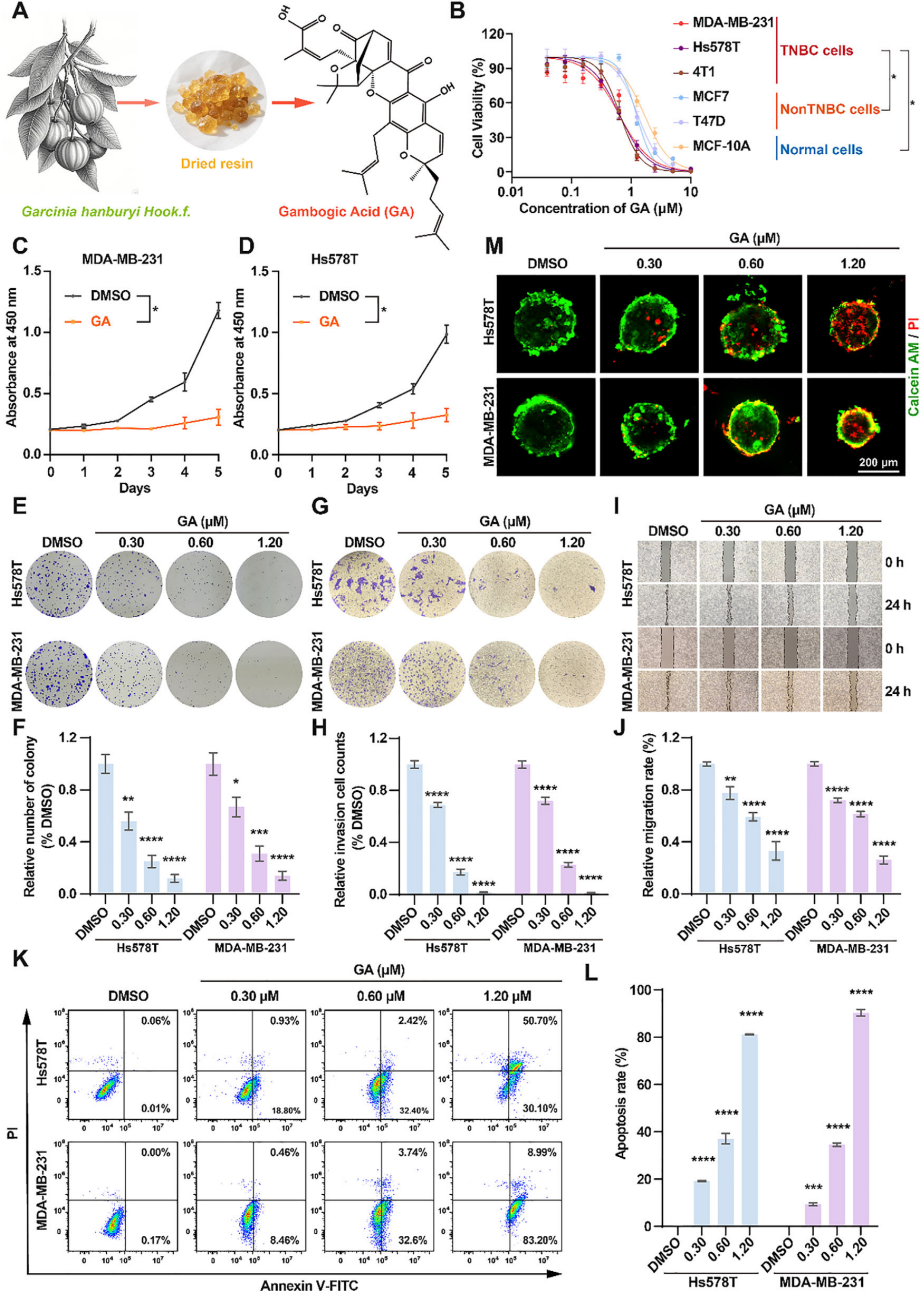
Gambogic acid effectively suppresses TNBC cell proliferation in vitro. (A) Chemical structure of GA, a compound derived from the dried resin of Garcinia hanburyi. (B) Dose‐response curves for cell viability in non‐TNBC cell lines (MCF‐10A, MCF‐7, T47D) and TNBC cell lines (MDA‐MB‐231, Hs578T, 4T1) following GA treatment. (C,D) Proliferation of TNBC cells treated with GA (0.6 µm) or DMSO control, as measured by the CCK‐8 assay. (E,F) Representative images and quantitative analyses of TNBC cell colony formation following treatment with increasing concentrations of GA. (G,H) Representative images and quantitative analyses of TNBC cell invasion assays following treatment with increasing concentrations of GA. (I,J) Representative images and quantitative analyses of TNBC cell migration assays following treatment with increasing concentrations of GA. (K,L) Apoptosis in TNBC cells treated with varying concentrations of GA, quantified by flow cytometry. The apoptosis rate was calculated as the sum of early apoptotic cells (Annexin V^+^/PI^−^) and late apoptotic cells (Annexin V^+^/PI^+^). (M) Representative fluorescence images of TNBC three‐dimensional multicellular tumor spheroids observed under laser confocal microscopy following treatment with different concentration gradients of GA or DMSO. Live cells were labelled with Calcein‐AM (green fluorescence), while dead cells were labelled with PI (red fluorescence). Data were presented as the mean ± SEM (*n* = 3). Statistical significance relative to the DMSO control was determined by one‐way ANOVA. Significance levels are indicated as ^*^ *p* < 0.05, ^**^ *p* < 0.01, ^***^ *p* < 0.001, and ^****^ *p* < 0.0001.

GA, a natural compound derived from the dried resin of *Garcinia hanburyi Hook. f*. (Figure 1A), has been documented to exhibit both anti‐inflammatory activity and anticancer potential [25, 28, 29]. However, its efficacy and mechanism of action specifically against TNBC remain inadequately characterized. To evaluate the anti‐TNBC potential of GA, we first assessed its cytotoxicity across a panel of breast cancer cell lines. Using the Cell Counting Kit‐8 (CCK‐8) assay, we determined the half‐maximal inhibitory concentration (IC_50_) of GA. Notably, GA displayed significantly greater cytotoxicity in TNBC cell lines (4T1, MDA‐MB‐231, Hs578T; IC_50_< 1 µm) compared to non‐TNBC lines, such as normal mammary epithelial cells (MCF‐10A) and estrogen receptor‐positive (ER^+^) breast cancer cells MCF‐7 and T47D (Figure 1B; Figure S1). This pronounced selectivity toward TNBC cells prompted further investigation into its anti‐cancer activity in vitro.

We next evaluated the impact of GA on key oncogenic phenotypes. GA treatment significantly inhibited TNBC cell proliferation (MDA‐MB‐231, Hs578T) (Figure 1C,D). In addition, GA markedly suppressed colony formation, indicating a loss of long‐term proliferative potential and clonogenicity in a dose‐dependent manner (Figure 1E,F). GA also markedly suppressed both cellular migration and invasive capacity, as evidenced by wound‑healing assays and Transwell Matrigel invasion experiments, respectively(Figure 1G–J). To better recapitulate the tumor microenvironment and evaluate cytotoxicity under more physiologically relevant conditions, we employed three‐dimensional multicellular tumor spheroids (MCTS) [33]. Administration of GA produced a pronounced decrease in spheroid size along with elevated cell mortality, as revealed by intense red fluorescence from propidium iodide (PI) staining of nonviable or dying cells, in contrast to the mainly green calcein‑AM signal characteristic of live cells in the control group (Figure 1M). Collectively, these findings demonstrate that GA exerts potent anti‐proliferative and cytotoxic effects against TNBC cells and tumor‐like structures in vitro.

### 2.2 GA Potently Inhibits TNBC Tumor Growth and Pulmonary Metastasis in Vivo

**FIGURE 2:**
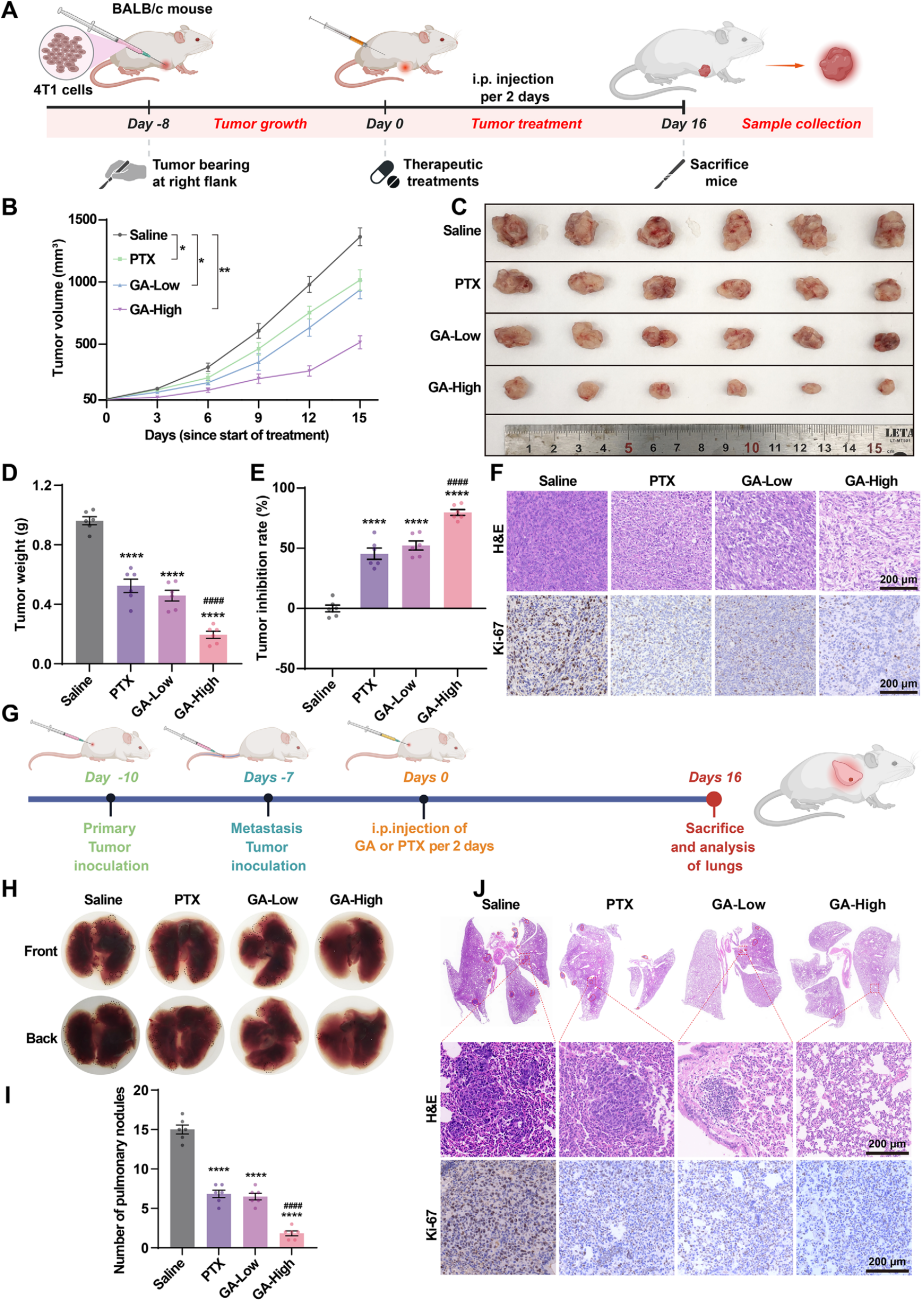
GA potently inhibits TNBC tumor growth and pulmonary metastasis in vivo. (A) Schematic illustration of the treatment regimen in 4T1 tumor‐bearing BALB/c mice. (B) Tumor growth curves recorded throughout the treatment period. (C) Representative images of excised tumors. (D,E) Endpoint tumor weights and corresponding tumor inhibition rates. (F) Histopathological evaluation of tumor sections collected on day 15 by H&E staining and Ki‐67 immunohistochemistry. (G) Schematic representation of treatment regimens administered to BALB/c mice bearing both primary and metastatic tumors. (H,I) Representative images of lung metastases (indicated by red circles) and corresponding quantitative analyses. (J) H&E and Ki‐67 staining of lung sections. Data were presented as the mean ± SEM (*n* = 6). Statistical significance versus the saline control or PTX was determined by one‐way ANOVA. Significance levels were indicated as ^****^ *p* < 0.0001 versus control; ^####^ *p* < 0.0001 versus PTX.

To confirm the *in vitro* therapeutic potential of GA, a syngeneic orthotopic breast tumor model was created by implanting murine TNBC 4T1 cells into the mammary fat pads of BALB/c mice (Figure 2A). Upon tumor establishment, mice were allocated into four experimental cohorts: a vehicle‐treated control group receiving saline (saline), paclitaxel (PTX, 10 mg/kg; a standard chemotherapeutic for TNBC), low‐dose GA (2 mg/kg), or high‐dose GA (6 mg/kg), administered via intraperitoneal injection. GA treatment resulted in a potent, dose‐dependent suppression of primary tumor growth. Tumor volume measurements over the treatment course (Figure 2B) and final tumor weight at the study endpoint (Figure 2C,D) showed that GA suppressed tumor growth in a dose‑dependent manner relative to the vehicle‑treated control group. Notably, high‐dose GA demonstrated superior antitumor efficacy compared to PTX (Figure 2E), underscoring its viability as an attractive candidate for therapeutic development. Histopathological and immunohistochemical analyses further corroborated these findings. Staining of excised tumor tissue sections with hematoxylin and eosin (H&E) (Figure 2F) demonstrated densely packed nuclei and preserved architecture in vehicle‐treated tumors, whereas tumors from PTX‐ and GA‐treated mice exhibited reduced cellularity, nuclear pyknosis, and widespread necrosis, with the GA‑High group showing the greatest cytotoxic impact. Immunohistochemistry for the proliferation marker Ki‐67 [34] showed a marked decline in Ki‑67‐positive cells in both PTX‐ and GA‐treated tumors, consistent with the observed growth inhibition (Figure 2F).

Given the aggressive metastatic nature of TNBC, particularly its strong predilection for pulmonary dissemination, we next assessed GA's anti‐metastatic efficacy using an experimental lung metastasis model (Figure 2G). Mice bearing orthotopic 4T1 tumors received intravenous injections of luciferase‐expressing 4T1 cells and were treated according to the same regimen. GA significantly reduced lung metastatic burden in a dose‐dependent manner. Bioluminescence imaging (Figure 2H), enumeration of visible lung surface nodules (Figure 2I), and histological analysis of H&E‐stained lung sections, as well as immunohistochemistry analysis of Ki‐67 (Figure 2J), consistently demonstrated decreased metastatic foci in GA‐treated mice compared to controls. Importantly, GA‐High exhibited greater anti‐metastatic activity than PTX. Together, these in vivo results demonstrate that GA effectively suppresses both primary tumor growth and pulmonary metastasis in TNBC, offering strong preclinical support for its potential development as a treatment for this highly aggressive cancer type.

### 2.3 Chemoproteomic Profiling Identifies SHMT2 as a Principal Covalent Binding Target of GA in TNBC Cells

**FIGURE 3:**
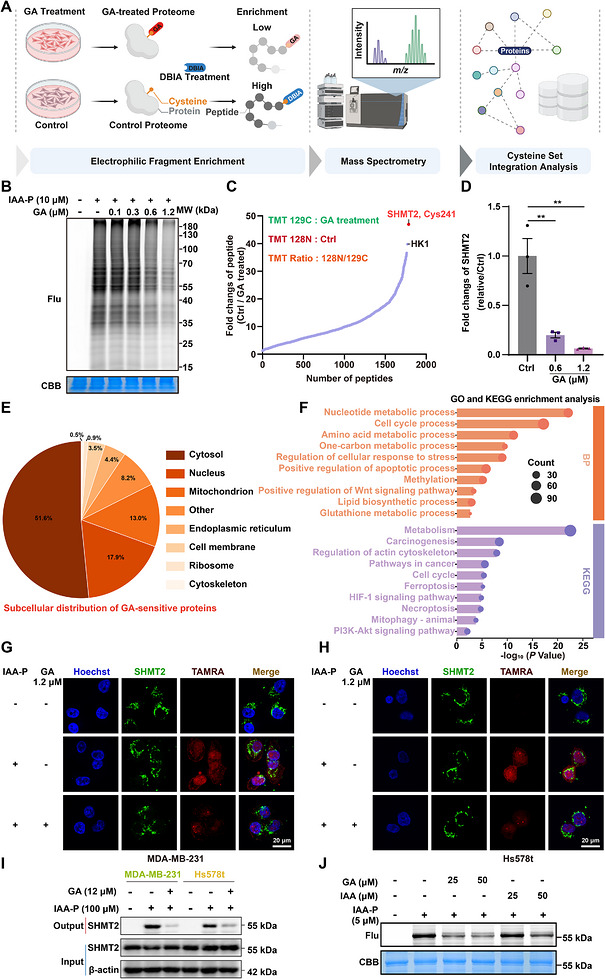
Chemoproteomic profiling identifies SHMT2 as a primary covalent target of GA in TNBC cells. (A) Overall workflow for identifying GA targets using activity‐based chemical proteomic profiling of cysteine reactivity. (B) In situ competition of protein labeling with IAA‐P by GA in TNBC cells. Flu: fluorescence image; CBB: Coomassie Brilliant Blue staining. (C) Identification of potential GA targets in TNBC cells through chemical proteomic profiling. (D) Competitive effects of increasing GA concentrations on the binding of SHMT2 to IAA. (E) Subcellular distribution of GA‐sensitive proteins. (F) Bioinformatic analysis of GA‐sensitive proteins. (G,H) GA reduces SHMT2 binding to IAA‐P, as shown by immunofluorescence in TNBC cells. TAMRA: tetramethyl rhodamine azide. (I) Validation of the GA‐SHMT2 interaction via pull‐down and Western blot assay. (J) GA competes with IAA‐P for binding to recombinant human SHMT2 (rhSHMT2) protein in an in‐gel fluorescence assay. Data were presented as the mean ± SEM (*n* = 3); ^**^ *p* < 0.01 versus Ctrl.

GA functions as an electrophilic Michael acceptor owing to its α, β‐unsaturated carbonyl moiety, which enables covalent modification of nucleophilic cysteine residues in target proteins [32]. Given the essential function of reactive cysteine residues within enzyme catalytic domains and their participation in diverse cellular signaling mechanisms [35], we hypothesized that GA's potent anti‐TNBC activity may arise from covalent engagement with key functional cysteinyl thiols. To comprehensively profile the landscape of GA‐susceptible cysteines and identify the molecular targets underlying its efficacy, we employed a cysteine‐reactivity‐based competitive chemoproteomic strategy [36] (Figure 3A). Pre‐treatment of TNBC cells in situ with GA resulted in a dose‐dependent decrease in fluorescence following subsequent labeling with the cysteine‐specific probe iodoacetamide‐alkyne (IAA‐P) (Figure 3B; Figure S2A), indicating that GA effectively competes with the probe for cysteine modification.

We next conducted quantitative chemoproteomic profiling using the desthiobiotin‐iodoacetamide (DBIA) probe. Proteins from GA‐treated or control TNBC cells were subjected to in situ DBIA labeling, after which they were enriched, digested with trypsin, tagged with tandem mass tags (TMT), and analyzed by LC‐MS/MS (Figure 3A; Figure S2B). This approach generated a global map of GA‐sensitive cysteine residues (Figure 3C). Proteins displaying reduced DBIA labeling in GA‐treated samples, reflected by high control‐to‐GA (Ctrl/GA) intensity ratios, were prioritized as potential covalent targets. Among these candidates, SHMT2 was selected for further investigation based on its identification as a high‐confidence target (supported by a high control‐to‐GA ratio and dose‐dependent behavior) and biological relevance. SHMT2 emerged as the top‐ranked candidate based on both the magnitude of competitive reduction and its dose‐responsive behavior (Figure 3D), strongly implicating it as a primary covalent target of GA in TNBC cells. Bioinformatic analysis of the GA‐sensitive proteome further contextualized these findings. Subcellular localization data indicated an enrichment of GA targets in the nucleus, cytoplasm, and mitochondria (Figure 3E). Analysis through the Gene Ontology (GO) and Kyoto Encyclopedia of Genes and Genomes (KEGG) databases revealed significant enrichment in pathways linked to cellular metabolism, particularly amino acid and one‐carbon metabolism (Figure 3F; Figure S2C). As a pivotal mitochondrial enzyme in one‐carbon metabolism, SHMT2 was highly consistent with the subcellular localization and pathway enrichment patterns identified in our chemoproteomic dataset. Moreover, previous studies have implicated SHMT2 in breast cancer and other malignancies [18, 19]. Together, these observations highlighted SHMT2 as a compelling candidate for subsequent mechanistic investigation.

We next conducted orthogonal biochemical assays to confirm the direct interaction between GA and SHMT2. Immunofluorescence colocalization studies revealed strong overlap between IAA‐P labeling (indicative of reactive cysteines) and endogenous SHMT2 in TNBC cells. Notably, GA pre‐treatment substantially reduced this colocalized signal (Figure 3G,H), suggesting direct competition at the cellular level. Cellular pull‐down assays using the DBIA probe under competitive conditions (GA + DBIA vs. DBIA alone) showed a marked reduction in SHMT2 enrichment upon GA treatment (Figure 3I), further confirming GA's covalent engagement of SHMT2 in situ. To definitively demonstrate direct binding, we expressed and purified recombinant human SHMT2 (rhSHMT2). In vitro IAA‐P labeling assays revealed that GA effectively competed for labeling of rhSHMT2 (Figure 3J). Moreover, pre‐incubation with iodoacetamide (IAA), a canonical cysteine alkylator, also dose‐dependently inhibited IAA‐P labeling of rhSHMT2 (Figure 3J), confirming that GA modifies cysteine residues on SHMT2. Importantly, we further validated the structural differences between SHMT1 and SHMT2 using sequence alignment and pull‑down Western blot assays. These analyses demonstrated that GA did not bind to SHMT1 but instead forms a covalent bond with SHMT2, thereby highlighting the specificity of the GA‐SHMT2 interaction (Figure S10). Collectively, these integrated chemoproteomic and biochemical data robustly identify and validate SHMT2 as a direct, covalent cellular target of GA in TNBC cells.

### 2.4 GA Covalently Modifies Cys241 to Inhibit SHMT2 Catalytic Activity and Oligomerization

**FIGURE 4:**
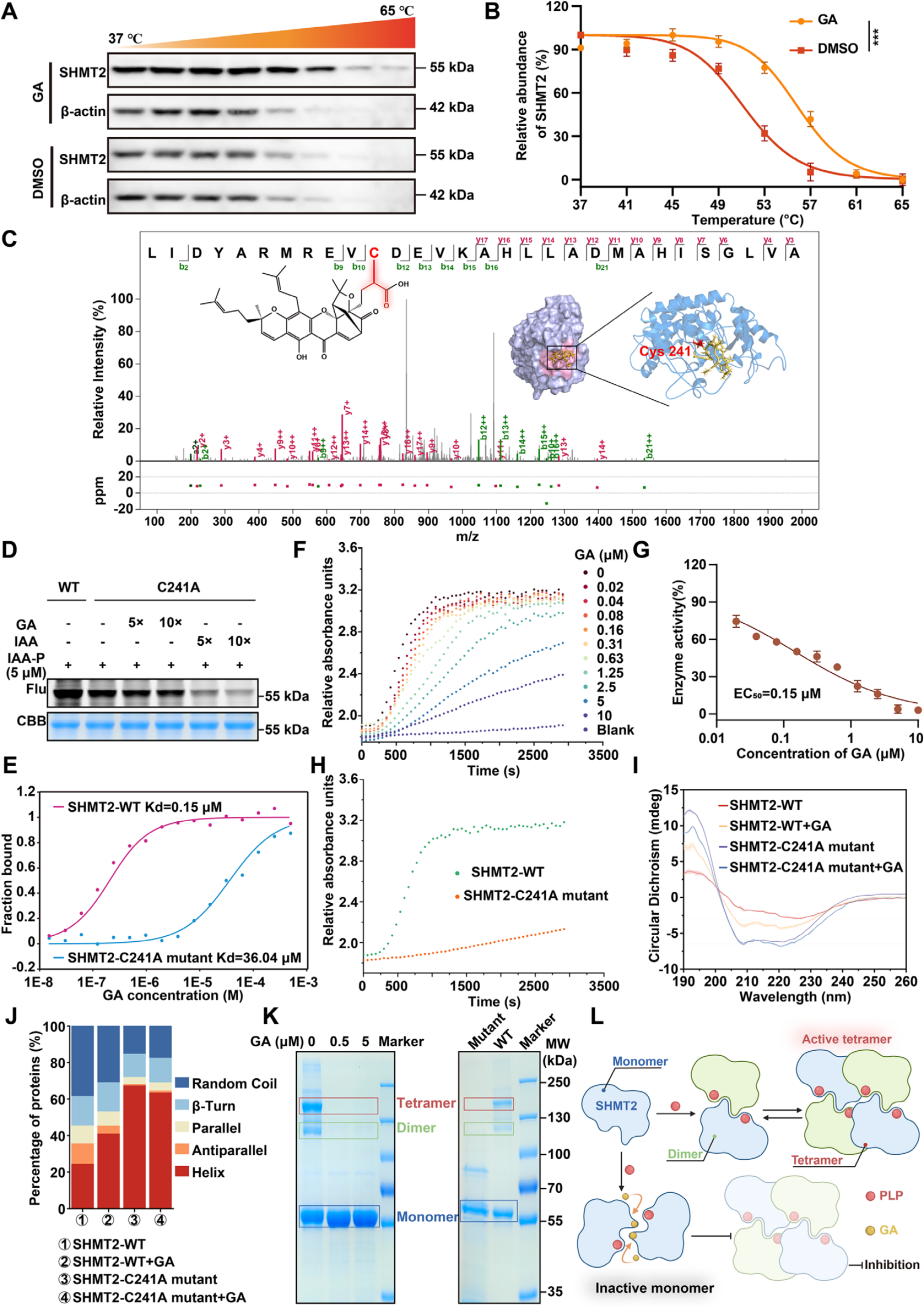
GA covalently modifies Cys241 to inhibit SHMT2 catalytic activity and oligomerization. (A,B) CETSA‐Western blot (WB) assay (A) and quantitative analysis (B) demonstrating GA binding to SHMT2. (C) Identification of the GA binding site on SHMT2 by LC‐MS/MS; molecular docking reveals that GA forms a covalent bond with Cys241 of SHMT2. (D) IAA‐P labeling of recombinant human SHMT2 (rhSHMT2) and its mutant SHMT2‐C241A in an in‐gel fluorescence assay. (E) Binding affinity of GA to rhSHMT2 and its mutants, determined by microscale thermophoresis (MST). (F) GA markedly inhibits SHMT2 enzymatic activity. (G) Half‐maximal inhibitory concentration (EC_50_) of GA for SHMT2 enzymatic activity. (H) C241 mutation significantly reduces SHMT2 enzymatic activity. (I,J) Circular dichroism (I) and secondary structure quantification (J) of SHMT2 and its mutants in the presence or absence of GA. (K) GA treatment and C241 mutation significantly impair SHMT2 tetramer formation. (L) Schematic model illustrating GA's covalent binding to SHMT2 at Cys241, thereby preventing tetramer assembly and markedly inhibiting enzymatic activity. Data were presented as the mean ± SEM (*n* = 3); ^***^ *p* < 0.001 versus DMSO.

Having identified SHMT2 as a direct covalent target of GA, we next sought to pinpoint the specific modification site and elucidate the structural and functional consequences of GA binding. To assess the binding affinity and stability of the GA‐SHMT2 complex within cells, we applied the cellular thermal shift assay (CETSA). Treatment with GA markedly elevated SHMT2's thermal stability, as indicated by enhanced resistance to heat‐induced denaturation compared to vehicle‐treated controls across a temperature gradient (Figure 4A,B). This GA‐dependent stabilization supports the formation of a stable GA‐SHMT2 complex in situ. To identify the precise cysteine residue targeted by GA, we conducted LC‐MS/MS analysis on recombinant human SHMT2 (rhSHMT2) incubated with GA. This analysis unambiguously identified Cys241 as the primary site of covalent adduction (Figure 4C). Molecular docking simulations corroborated this result, showing that the electrophilic carbon of GA's α, β‐unsaturated carbonyl moiety is optimally positioned to form a covalent linkage with the thiol moiety of Cys241 (Figure 4C). To functionally validate the role of Cys241 in GA binding, we generated a Cys241‐to‐alanine mutant of SHMT2 (SHMT2‐C241A) and assessed its reactivity. In vitro labeling with the IAA‐P showed markedly reduced signal for SHMT2‐C241A compared to wild‐type (WT) SHMT2 (Figure 4D), confirming that Cys241 is a highly reactive site. Importantly, GA failed to compete with IAA‐P labeling of the SHMT2‐C241A mutant, in contrast to its potent competition for WT SHMT2 (Figure 4D). Binding affinity measured by microscale thermophoresis (MST) revealed a strong interaction between GA and WT SHMT2 (K_D_ = 0.15 µm), which was dramatically weakened in the C241A mutant (K_D_ = 36.04 µm) (Figure 4E), conclusively establishing Cys241 as the principal residue mediating GA binding.

We next examined the functional impact of GA binding and Cys241 mutation on SHMT2 enzymatic activity. Exposure of rhSHMT2 to GA produced a dose‑responsive suppression of its enzymatic activity, yielding an EC_50_ value of 0.15 µm (Figure 4F,G). Additionally, GA treatment did not alter SHMT2 expression (Figure S3A,B). Notably, the SHMT2‐C241A mutant exhibited severely compromised catalytic activity, nearly abolishing its ability to catalyze the conversion of serine to glycine (Figure 4H), underscoring the essential role of Cys241 in SHMT2 function. To investigate the structural basis of this inhibition, we performed circular dichroism (CD) spectroscopy. Both GA binding and the C241A mutation led to significant alterations in SHMT2's secondary structure, characterized by increased helical content and decreased β‐sheet and random coil elements (Figure 4I,J). Given that SHMT2 requires pyridoxal phosphate (PLP)‐dependent oligomerization from an inactive monomer to an active tetramer [19], we hypothesized that perturbation of Cys241 disrupts this transition. Chemical cross‐linking assays using disuccinimidyl suberate (DSS) confirmed this hypothesis: while WT SHMT2 efficiently formed tetramers, both GA‐bound WT and SHMT2‐C241A predominantly remained in the monomer state (Figure 4K). Collectively, these findings establish a comprehensive mechanism by which GA covalently modifies the functionally critical Cys241 residue of SHMT2. This covalent interaction induces conformational changes, impairs the PLP‐dependent monomer‐to‐tetramer transition, and ultimately abolishes SHMT2 enzymatic activity (Figure 4L).

### 2.5 Global Proteomic Profiling Implicates Mitochondrial Dysfunction and Ferroptosis as Key Downstream Effectors of GA‐Mediated SHMT2 Inhibition

**FIGURE 5:**
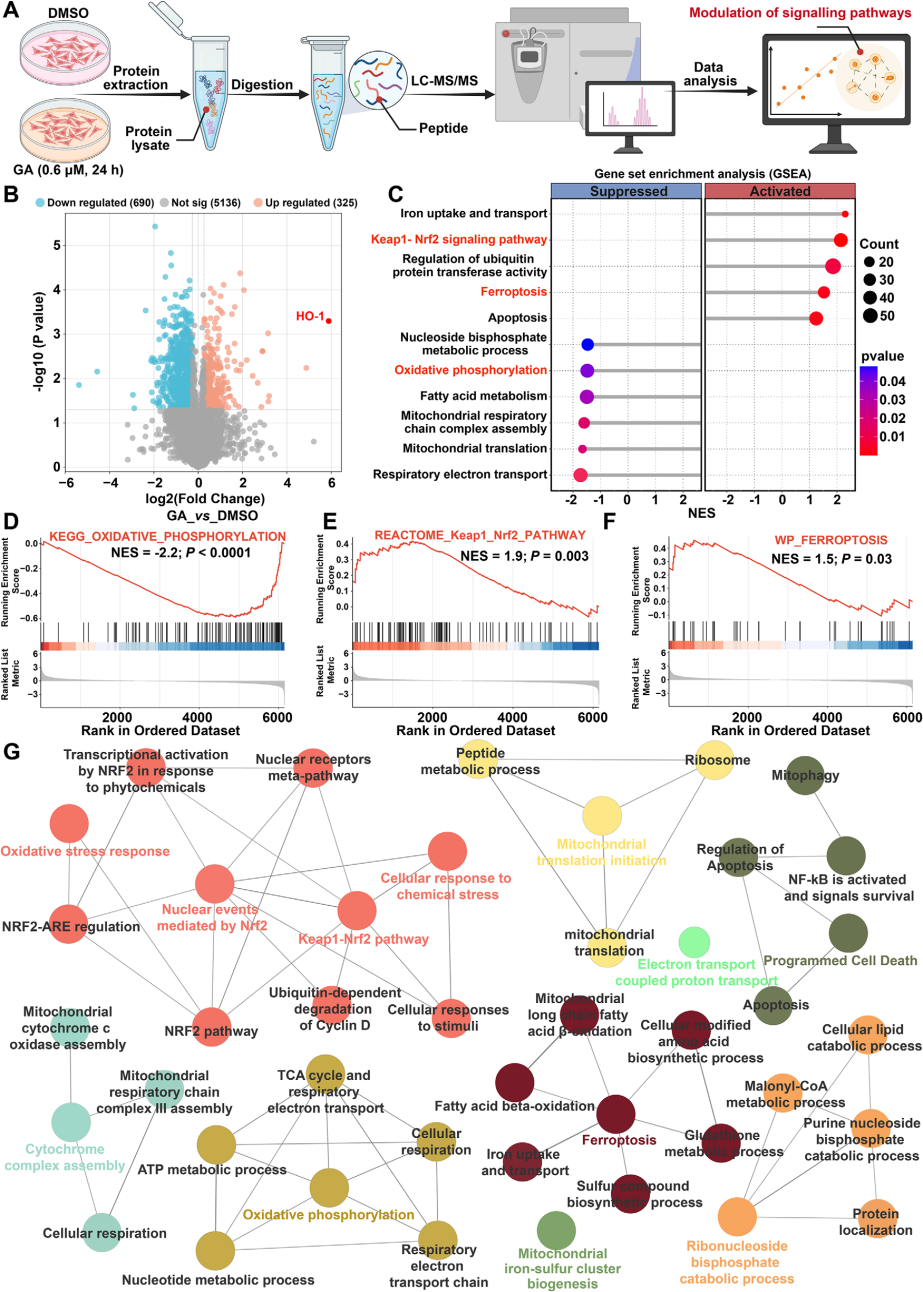
Global proteomic profiling implicates mitochondrial dysfunction and ferroptosis as key downstream effectors of GA‐mediated SHMT2 inhibition. (A) Overall workflow of proteomic analysis of TNBC cells treated with GA. (B) Volcano plot showing differentially expressed proteins (DEPs) between the GA treatment group and the DMSO control group (pink: upregulated; grey: unchanged; blue: downregulated). (C) GSEA identifying multiple signaling pathways regulated by GA in TNBC cells. (D,F) GA significantly inhibited oxidative phosphorylation (D), activated the Keap1‐Nrf2 signaling pathway (E), and induced ferroptosis (F) in TNBC cells. (G) GO and KEGG analyses revealing that GA inhibits TNBC progression through the regulation of multiple signaling pathways.

Having established that GA covalently inhibits SHMT2, we next sought to elucidate the global cellular response and identify key downstream pathways mediating its anti‐TNBC effects. To this end, we performed quantitative global proteomic analysis on MDA‐MB‐231 TNBC cells exposed to GA or vehicle control (Figure 5A). This analysis identified 6,151 proteins, with differential expression revealing widespread proteomic remodeling following GA treatment: 690 proteins were significantly downregulated, while 325 were significantly upregulated relative to controls (Figure 5B). Notably, heme oxygenase‐1 (HO‐1) emerged as the most prominently upregulated protein, showing a ∼60‐fold increase in abundance, strongly implicating the HO‐1‐associated regulatory axis in GA's mechanism of action. To systematically interpret these proteomic alterations, we conducted gene set enrichment analysis (GSEA). This unbiased approach revealed that GA treatment led to robust suppression of mitochondrial metabolic pathways essential for energy production, including nucleoside bisphosphate metabolism, oxidative phosphorylation, and mitochondrial respiration (Figure 5C). Conversely, GA significantly activated cellular stress response pathways, particularly the Keap1‐Nrf2 signaling axis, ferroptosis, and apoptosis. Enrichment plots confirmed the marked downregulation of oxidative phosphorylation (Figure 5D), robust stimulation of the Keap1‐Nrf2 signaling cascade (Figure 5E), and significant induction of ferroptosis (Figure 5F) in GA‐treated cells.

Proteomic clustered heat maps revealed pronounced elevation in Nrf2 and its downstream effectors, including HO‑1 and NQO1, alongside a decreased expression of Keap1, which serves as the primary negative regulator of Nrf2 (Figure S4A,B). Furthermore, significant reductions were observed in essential elements of the mitochondrial electron transport chain, including NDUFA2, which supports the proteomic data indicating compromised oxidative phosphorylation. Integrated pathway analysis of the 1,015 significantly altered proteins using GO and KEGG enrichment further reinforced these findings. Enriched biological processes and pathways closely aligned with the GSEA results and included oxidative phosphorylation, mitochondrial respiration, ribonucleotide biosynthesis, oxidative stress response, ferroptosis, and the Keap1‐Nrf2 pathway (Figure 5G). Together, this integrated proteomic and functional analysis supports a mechanistic model in which GA‐mediated SHMT2 inhibition induces mitochondrial dysfunction, activates the Nrf2/HO‐1 stress axis, and ultimately triggers ferroptosis. We next conducted targeted mechanistic studies to experimentally validate and dissect this signaling cascade.

### 2.6 SHMT2 Inhibition by GA Induces Comprehensive Mitochondrial Dysfunction in TNBC Cells

**FIGURE 6:**
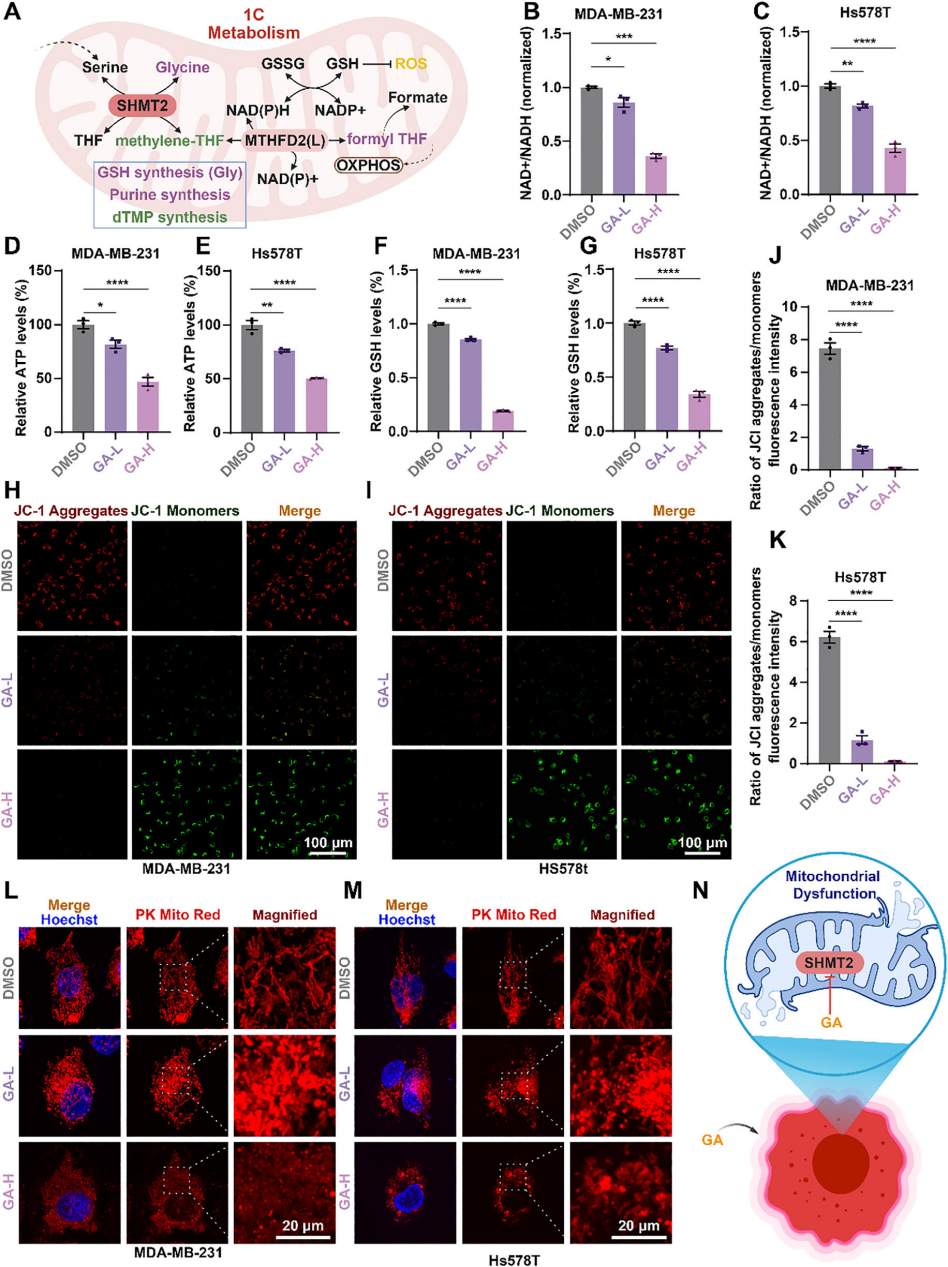
SHMT2 inhibition by GA induces comprehensive mitochondrial dysfunction in TNBC cells. (A) Schematic diagram illustrating the role of SHMT2 in key mitochondrial metabolic processes. (B,C) GA significantly reduced the NAD^+^/NADH ratio in TNBC cells. (D,E) GA significantly decreased ATP levels in TNBC cells. (F,G) GA significantly reduced GSH levels in TNBC cells. (H–K) Assessment of mitochondrial membrane potential in TNBC cells following GA treatment. (L,M) Analysis of mitochondrial morphology in TNBC cells after GA treatment. (N) Schematic diagram depicting GA‐mediated covalent inhibition of SHMT2, leading to irreversible mitochondrial morphofunctional collapse in TNBC cells. Data were presented as means ± SEM (*n* = 3). Statistical significance versus the DMSO control was determined by one‐way ANOVA. Significance levels are indicated as ^*^ *p* < 0.05, ^**^ *p* < 0.01, ^***^ *p* < 0.001, and ^****^ *p* < 0.0001.

SHMT2 functions as a central regulator of mitochondrial one‐carbon metabolism, directing serine catabolism to support nucleotide biosynthesis, maintain redox homeostasis through GSH production, and provide one‐carbon units essential for mitochondrial respiration and energy generation [18, 22, 37] (Figure 6A). Building on our proteomic evidence implicating mitochondrial dysfunction, we hypothesized that GA‐mediated covalent inhibition of SHMT2 disrupts these critical metabolic pathways, leading to profound and irreversible mitochondrial compromise. To test this, we performed a series of targeted assays assessing mitochondrial health and function in GA‐treated TNBC cells. First, we evaluated mitochondrial redox status by measuring the NAD^+^/NADH ratio. GA treatment caused a significant decrease in this ratio (Figure 6B,C), indicating a shift toward a more reduced mitochondrial environment and impaired oxidative metabolism. Consistent with this, intracellular ATP quantification revealed a severe, dose‐dependent reduction in ATP levels following GA exposure (Figure 6D,E), reflecting impaired oxidative phosphorylation and supporting the proteomic finding of reduced electron transport chain (ETC) subunit expression (Figure S4A,B). Given SHMT2's critical role in supplying glycine for GSH biosynthesis [37], we next measured intracellular GSH content, finding a marked depletion in GA‐treated TNBC cells (Figure 6F,G), directly linking SHMT2 inhibition to the collapse of the primary mitochondrial antioxidant defense.

The assessment of mitochondrial membrane potential (ΔΨm) was performed using the JC‑1 probe. In healthy mitochondria, JC‑1 aggregates emit red fluorescence, whereas a loss of ΔΨm shifts the signal to green fluorescence from monomers. GA exposure caused a pronounced, concentration‑dependent dissipation of ΔΨm, evidenced by an elevated green/red fluorescence ratio (Figure 6H–K), signifying disruption of the proton gradient essential for ATP production. To visualize the structural impact of mitochondrial dysfunction, confocal microscopy using the mitochondrial‐specific dye PK Mito Red was performed. Control cells displayed an extensive, interconnected mitochondrial network, whereas GA‐treated cells exhibited pronounced mitochondrial fragmentation, characterized by punctate, shortened organelles and loss of normal tubular architecture (Figure 6L,M). Significantly, GA treatment markedly suppressed the OCR levels in TNBC cells, including reductions in both basal and maximal respiration (Figure S4C,D). Collectively, these multiparametric analyses demonstrate that GA, through covalent inhibition of SHMT2, precipitates catastrophic mitochondrial morphofunctional collapse in TNBC cells (Figure 6N).

### 2.7 Mitochondrial Dysfunction Driven by GA Activates the Nrf2/HO‐1 Axis, Culminating in Iron Overload‐Dependent Ferroptosis

**FIGURE 7:**
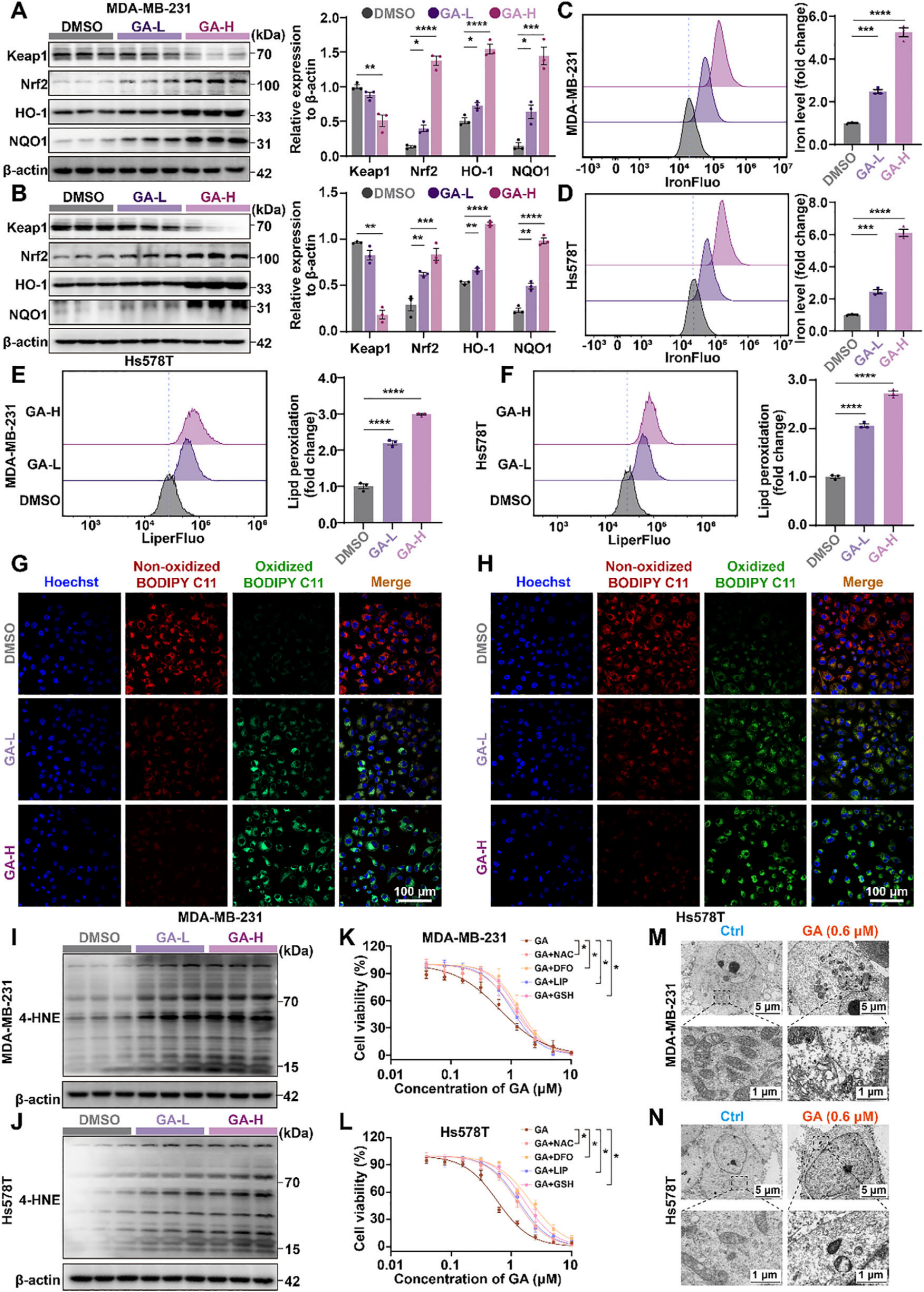
Mitochondrial dysfunction driven by GA activates the Nrf2/HO‐1 axis, culminating in iron overload‐dependent ferroptosis. (A,B) Western blot analysis of Keap1, Nrf2, HO‐1, and NQO1 expression in TNBC cells before and after GA treatment. (C,D) GA increases Fe^2+^ levels in TNBC cells in a dose‐dependent manner. (E,F) GA elevates lipid peroxidation levels in TNBC cells in a dose‐dependent manner. (G,H) Confocal microscopy showing changes in lipid peroxidation levels in TNBC cells following GA treatment. Scale bar = 100 µm. (I,J) Expression levels of 4‐HNE in TNBC cells before and after GA treatment. (K,L) TNBC cell death induced by GA is rescued by classical ferroptosis inhibitors. (M,N) Transmission electron microscopy images of TNBC cells with or without GA treatment. Scale bar = 1 µm or 5 µm. Data were presented as means ± SEM (*n* = 3). Statistical significance versus the DMSO control was determined by one‐way ANOVA. Significance levels are indicated as ^*^ *p* < 0.05, ^**^ *p* < 0.01, ^***^ *p* < 0.001, and ^****^ *p* < 0.0001.

Our integrated proteomic and mitochondrial functional analyses positioned mitochondrial collapse as a pivotal event that precedes activation of the Nrf2 pathway and engagement of ferroptosis. We hypothesized that mitochondrial stress induced by GA‐mediated SHMT2 inhibition serves as the primary trigger for Nrf2 activation, leading to HO‐1 upregulation, iron overload, and consequent ferroptosis. Western blot analysis confirmed dose‐dependent upregulation in Nrf2 protein and major downstream effectors–heme oxygenase‑1 (HO‑1) and NAD(P)H quinone dehydrogenase 1 (NQO1), in TNBC cells following GA treatment (Figure 7A,B), consistent with the profound HO‐1 induction observed in our proteomic screen. Although Nrf2 activation typically confers cytoprotection, HO‐1 exerts a dual role in ferroptosis. Remarkably, GA induced a rapid and robust surge in HO‐1 levels within TNBC cells, exhibiting a distinct time‐dependent pattern over a short timeframe (Figure S5A). By degrading heme, HO‐1 releases free ferrous iron (Fe^2+^), which can catalyze lipid peroxidation and promote ferroptosis under redox homeostasis imbalance. To directly assess intracellular Fe^2+^ accumulation, we employed the Fe^2+^‐specific fluorescent probe FerroOrange combined with flow cytometry. GA treatment induced a pronounced, concentration‑dependent rise in labile Fe^2^^+^ levels in TNBC cells (Figure 7C,D), establishing a mechanistic link between GA‐induced HO‐1 expression and intracellular iron overload.

Iron overload, particularly Fe^2+^, drives ferroptosis through Fenton chemistry by catalyzing the peroxidation of polyunsaturated fatty acids (PUFAs) within cell membranes. We evaluated lipid peroxidation, the biochemical hallmark of ferroptosis, using complementary approaches. Flow cytometric analysis with the lipid peroxidation sensor BODIPY C11 revealed a significant, dose‐dependent increase in oxidized probe fluorescence (shift from red to green) in GA‐treated cells (Figure 7E,F). Confocal microscopy further confirmed widespread lipid peroxidation, visualized as intense green fluorescence within GA‐treated TNBC cells (Figure 7G,H). Biochemical assays substantiated these findings by demonstrating marked elevations in the lipid peroxidation byproducts malondialdehyde (MDA) (Figure S5B, C) and 4‐hydroxynonenal (4‐HNE) (Figure 7I,J) following GA treatment. To definitively establish ferroptosis as the principal mode of GA‐induced cytotoxicity, we conducted pharmacological rescue experiments using established ferroptosis inhibitors: the iron chelator deferoxamine (DFO), the radical‐trapping antioxidant liproxstatin‐1 (Lip‐1), the GSH precursor N‐acetylcysteine (NAC), and exogenous GSH. All treatments significantly attenuated GA‐induced cell death in TNBC cells (Figure 7K,L), confirming the critical role of iron‐dependent lipid peroxidation in GA's lethal mechanism. Finally, an ultrastructural analysis using transmission electron microscopy (TEM) identified typical mitochondrial abnormalities associated with ferroptosis in cells treated with GA. These abnormalities included decreased mitochondrial size, increased membrane density, loss of cristae, and the rupture of the outer membrane, with no evidence of classical apoptotic morphology (Figure 7M,N). Additionally, we investigated the levels of lipid peroxidation markers and the expression of Nrf2/HO‐1 signaling pathway proteins in mouse tumor tissues (Figure S11). GA treatment significantly impaired total SHMT enzyme activity in tumor lysates in a dose‐dependent manner (Figure S11A) and markedly increased lipid peroxidation levels in mouse tumor tissues (Figure S11B–D). Regarding signaling mechanisms, IHC analysis confirmed in vivo activation of the Nrf2/HO‐1 pathway, manifested by significant upregulation of Nrf2 and HO‐1 proteins alongside reduced Keap1 expression (Figure S11C–G). Collectively, these findings indicated that the activation of the Nrf2/HO‐1 pathway, driven by mitochondrial dysfunction, leads to intracellular iron overload, which in turn promotes lethal lipid peroxidation and ferroptosis, constituting the terminal effector mechanism underlying GA's potent anti‐TNBC activity.

### 2.8 SHMT2 is Essential for GA‐Induced Ferroptosis and Represents a Critical Vulnerability in TNBC

**FIGURE 8:**
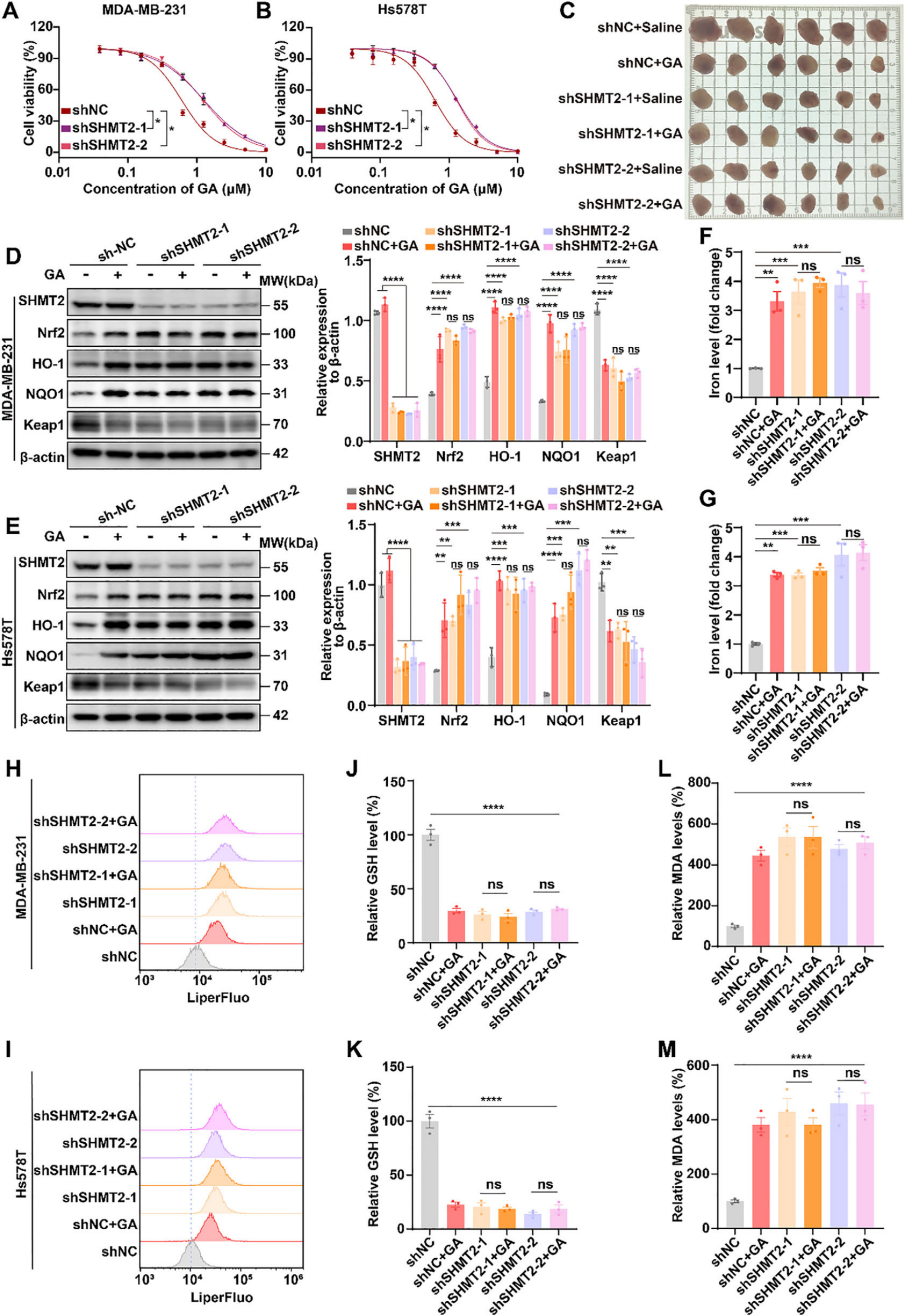
SHMT2 is essential for GA‐induced ferroptosis and represents a critical vulnerability in TNBC. (A,B) SHMT2 knockdown significantly reduces the sensitivity of TNBC cells to GA. (C) Representative picture of tumors derived from mice in the absence and presence of GA treatment, where tumors were formed by TNBC cells with SHMT2 knockdown. (D,E) SHMT2 knockdown markedly activates the Nrf2 signaling pathway in TNBC cells, regardless of GA treatment. (F,G) SHMT2 knockdown significantly increases ferrous ion levels in TNBC cells, independent of GA treatment. (H,I) Effects of SHMT2 knockdown and GA treatment on lipid peroxidation levels in TNBC cells. (J,K) Effects of SHMT2 knockdown and GA treatment on GSH levels in TNBC cells. (L,M) Effects of SHMT2 knockdown and GA treatment on MDA levels in TNBC cells. Data were presented as means ± SEM (*n* = 3). Significance levels are indicated as ^*^ *p* < 0.05, ^**^ *p* < 0.01, ^***^ *p* < 0.001, and ^****^ *p* < 0.0001.

While our preceding data established a mechanistic link between GA‐mediated SHMT2 inhibition and ferroptosis induction, we sought to definitively determine whether SHMT2 is functionally required for this cell death pathway. To this end, we generated stable SHMT2 knockdown (KD) TNBC cell lines (MDA‐MB‐231 and Hs578T) via lentiviral transduction of specific shRNA (Figure S6A,B). We initially evaluated how SHMT2 depletion influences GA sensitivity. Notably, SHMT2 KD markedly weakened GA's anti‑proliferative activity in both TNBC cell lines (Figure 8A,B). Conversely, SHMT2 overexpression increased sensitivity to GA in MCF‐7 and T47D, which are breast cancer cell lines with low endogenous SHMT2 (Figure S6C,D). Taken together, these data indicate that SHMT2 is not merely a target but is indispensable for mediating GA‐induced cytotoxicity. We further performed in vivo tumorigenesis assays to assess GA's effect in mice engrafted with SHMT2 KD TNBC cells. In mice bearing control shNC tumors, the administration of GA significantly reduced both the volume and weight of the tumors compared to the saline‐treated group (Figure 8C; Figure S6E–G). In contrast, in mice implanted with SHMT2 KD tumors, the inhibitory effect of GA was substantially diminished, with no significant differences in tumor parameters between GA‐ and saline‐treated groups (Figure 8C; Figure S6E–G). Given the pivotal role of the Nrf2/HO‐1 axis in GA‐triggered ferroptosis, we next examined this pathway's status in SHMT2 KD cells. Intriguingly, SHMT2 knockdown alone significantly activated the Nrf2 pathway, as evidenced by elevated expression of HO‐1 and NQO1 proteins (Figure 8D,E). This basal activation suggests that loss of SHMT2 intrinsically imposes cellular stress sufficient to engage this protective and executive signaling cascade. Importantly, GA treatment failed to further enhance Nrf2 pathway activation in SHMT2 KD cells (Figure 8D,E), implying that SHMT2 is the primary conduit through which GA engages this regulatory axis.

We then evaluated canonical ferroptosis markers in SHMT2 KD cells, with or without GA treatment. SHMT2 depletion alone induced a significant accumulation of labile Fe^2+^, reaching levels comparable to those observed in GA‐treated control cells (Figure 8F,G; Figure S7A,B). GA treatment did not further increase Fe^2+^ in SHMT2 KD cells. Flow cytometric analysis using BODIPY C11 revealed marked lipid peroxidation in SHMT2 KD cells, analogous to the extent induced by GA in control cells (Figure 8H,I; Figure S7C,D), with no additive effect upon GA treatment. Consistently, SHMT2 knockdown significantly elevated levels of MDA and 4‐HNE, lipid peroxidation byproducts, while reducing intracellular GSH content, mirroring the biochemical effects of GA treatment in controls (Figure 8J–M; Figure S7E,F). GA exerted no further impact on these ferroptosis indicators in SHMT2 KD cells. Collectively, these findings demonstrate that genetic ablation of SHMT2 phenocopies the ferroptosis effects induced by GA treatment. Moreover, the inability of GA to further potentiate ferroptosis or Nrf2 signaling in SHMT2 KD cells conclusively establishes SHMT2 as the critical, non‐redundant target mediating GA‐induced ferroptosis in TNBC. Crucially, our knockdown rescue experiments demonstrated that Cys241 site is indispensable for both SHMT2 activity and the survival of TNBC cells. This further reaffirmed that GA exerted its anti‐TNBC efficacy through a key mechanism of covalent binding to SHMT2 via Cys241 (Figure S8). This genetic evidence firmly underscores the central role of SHMT2 inhibition in GA's anti‐TNBC mechanism of action.

### 2.9 SHMT2 Overexpression is a Hallmark of TNBC and Promotes Tumor Progression by Suppressing Ferroptosis

**FIGURE 9:**
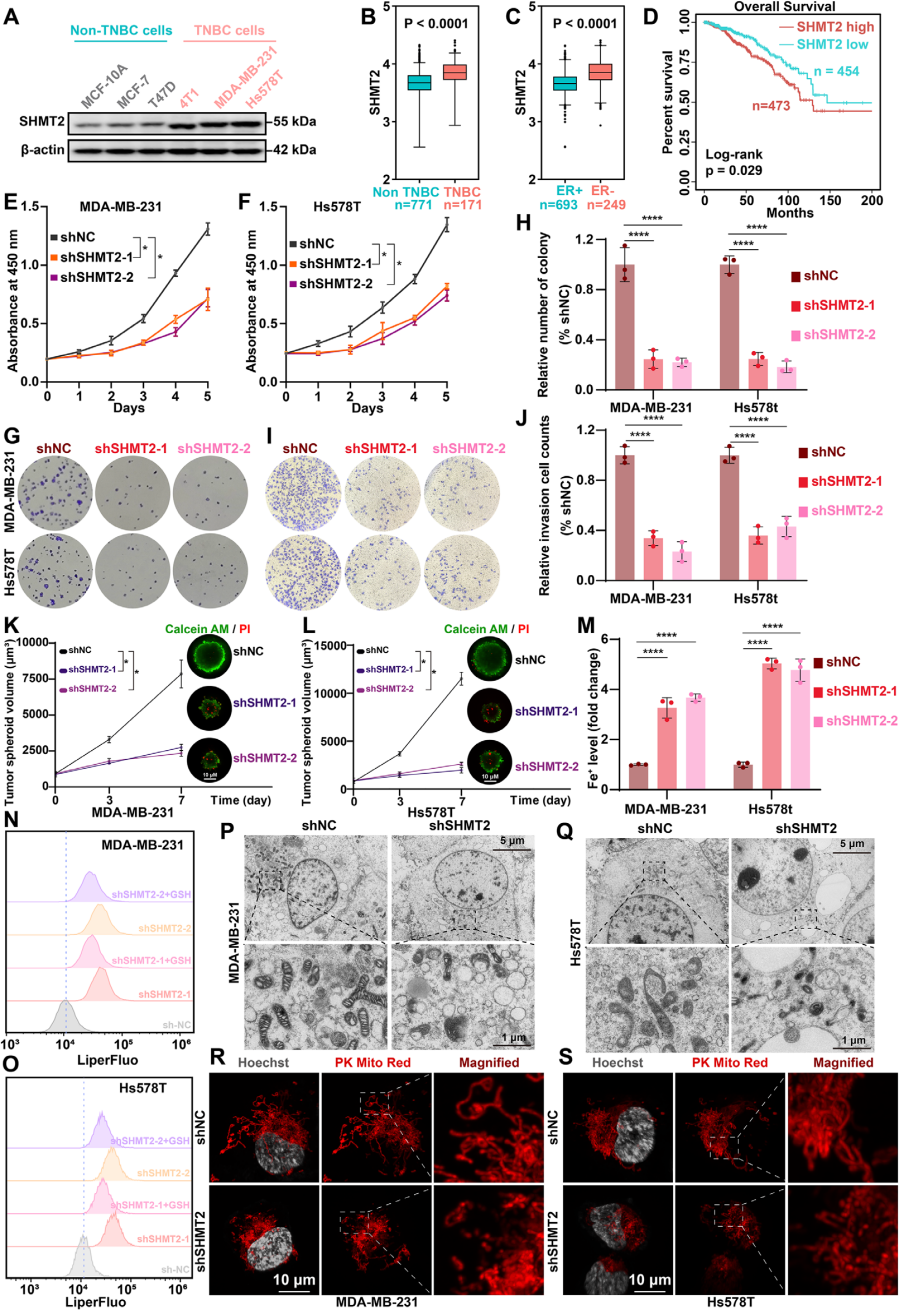
SHMT2 overexpression is a hallmark of TNBC and promotes tumor progression by suppressing ferroptosis. (A) Expression levels of SHMT2 protein in breast cancer cells across different subtypes. (B,C) SHMT2 expression in the triple‐negative subtype (B) and ER‐negative subtype (C) of breast cancer, analyzed using datasets from TCGA (B) and GSE5460 (C). (D) Overall survival analysis of breast cancer patients stratified by high versus low SHMT2 expression. (E,F) Proliferation of TNBC cells with SHMT2 knockdown measured by the CCK‐8 assay. (G,H) Proliferative capacity of TNBC cells after stable SHMT2 knockdown assessed by colony formation assay. (I,J) Representative images (I) and quantitative analysis (J) of TNBC cell invasion following stable SHMT2 knockdown. (K,L) Growth curves of multicellular tumor spheroids derived from wild‐type and stable SHMT2 knockdown TNBC cells. (M) Stable SHMT2 knockdown significantly increases ferrous iron levels in TNBC cells. (N,O) Stable SHMT2 knockdown markedly elevates lipid peroxidation levels in TNBC cells. (P,Q) Transmission electron microscopy (TEM) characterization of mitochondrial morphology and number in TNBC cells after stable SHMT2 knockdown. (R,S) Confocal microscopy analysis of mitochondrial morphological changes in TNBC cells following stable SHMT2 knockdown. Data were presented as means ± SEM (*n* = 3). Significance levels are indicated as ^*^ *p* < 0.05, ^**^ *p* < 0.01, ^***^ *p* < 0.001, and ^****^ *p* < 0.0001.

Our findings demonstrated that GA selectively targets TNBC cells and exerts its antitumor effects primarily via SHMT2 inhibition. This observation prompted us to investigate whether differential SHMT2 expression underlies the selective vulnerability of TNBC to GA. Moreover, the pronounced tumor‐suppressive effects observed following SHMT2 knockdown suggested a potential oncogenic role for SHMT2 in TNBC (Figure S6E–G). We first assessed SHMT2 expression across breast cancer subtypes. Western blot analysis revealed significantly elevated SHMT2 protein levels in multiple TNBC cell lines (MDA‐MB‐231, Hs578T, 4T1) compared with non‑TNBC counterparts, including normal mammary epithelial cells (MCF‑10A) and ER‑positive breast cancer lines (MCF‑7, T47D) (Figure 9A; Figure S9A). Consistently, bioinformatic interrogation of large clinical datasets (TCGA, GSE5460, METABRIC) confirmed significantly higher SHMT2 mRNA expression in TNBC and ER‐negative breast cancers compared to other subtypes (Figure 9B,C; Figure S9B).

Importantly, elevated SHMT2 expression correlates with poor patient prognosis. Survival analyses revealed that breast cancer patients exhibiting high SHMT2 expression experienced considerably reduced overall survival compared to those with lower expression levels (Figure 9D), establishing SHMT2 as a clinically relevant biomarker associated with aggressive disease phenotypes. To functionally characterize SHMT2's oncogenic role in TNBC, we utilized our previously established SHMT2 KD TNBC cell models (Figure S6A,B). Genetic depletion of SHMT2 markedly suppressed multiple oncogenic phenotypes. SHMT2 KD significantly inhibited cell proliferation (Figure 9E,F) and diminished colony‐forming capacity (Figure 9G,H). Furthermore, SHMT2‐deficient cells exhibited a pronounced reduction in invasive potential in Matrigel assays, a key feature underlying TNBC aggressiveness and metastatic propensity (Figure 9I,J). While SHMT2 KD did not impair the initial formation of multicellular tumor spheroids (MCTS), it severely compromised subsequent spheroid growth and expansion (Figure 9K,L), reflecting diminished tumorigenic capacity in a physiologically relevant three‐dimensional context. We next assessed intracellular Fe^2^^+^ levels in SHMT2 knockdown TNBC cells. The results demonstrated that SHMT2 knockdown significantly increased intracellular Fe^2+^ accumulation (Figure 9M; Figure S9C,D), indicating its essential role in maintaining iron homeostasis. Subsequent analysis revealed that SHMT2 knockdown induced lethal mitochondrial damage, characterized by collapsed membrane potential and a predominantly oxidized mitochondrial state. Notably, this loss of mitochondrial membrane potential was partially rescued by exogenous GSH supplementation, though not restored to baseline levels (Figure S9E–H). This observation suggests concurrent impairment of mitochondrial GSH biosynthesis upon SHMT2 knockdown, consistent with prior findings (Figure 6F,G). Correspondingly, SHMT2 knockdown markedly elevated lipid peroxidation in TNBC cells (Figure 9N,O; Figure S9I,J). Besides, knockdown of SHMT2 significantly reduced the intracellular OCR (Figure S9K). Finally, TEM and confocal imaging analyses showed that SHMT2 knockdown cells exhibited extensive mitochondrial fragmentation with disrupted tubular networks, manifesting as punctate and spherically swollen organelles compared to the continuous mitochondrial morphology in shNC controls (Figure 9P–S). Crucially, these collective alterations including iron dysregulation, mitochondrial damage, and lipid peroxidation, establish that SHMT2 loss‐of‐function induces ferroptosis in TNBC cells. Collectively, these data establish a paradigm in which SHMT2 overexpression functions as a metabolic safeguard in TNBC by preserving mitochondrial integrity and metabolic homeostasis, thereby actively suppressing ferroptosis and facilitating aggressive tumor progression. This oncogenic dependency on SHMT2 highlights its significance as a critical therapeutic vulnerability and validates it as the principal target mediating GA's potent anti‐TNBC activity.

## 3 Discussion

**FIGURE 10:**
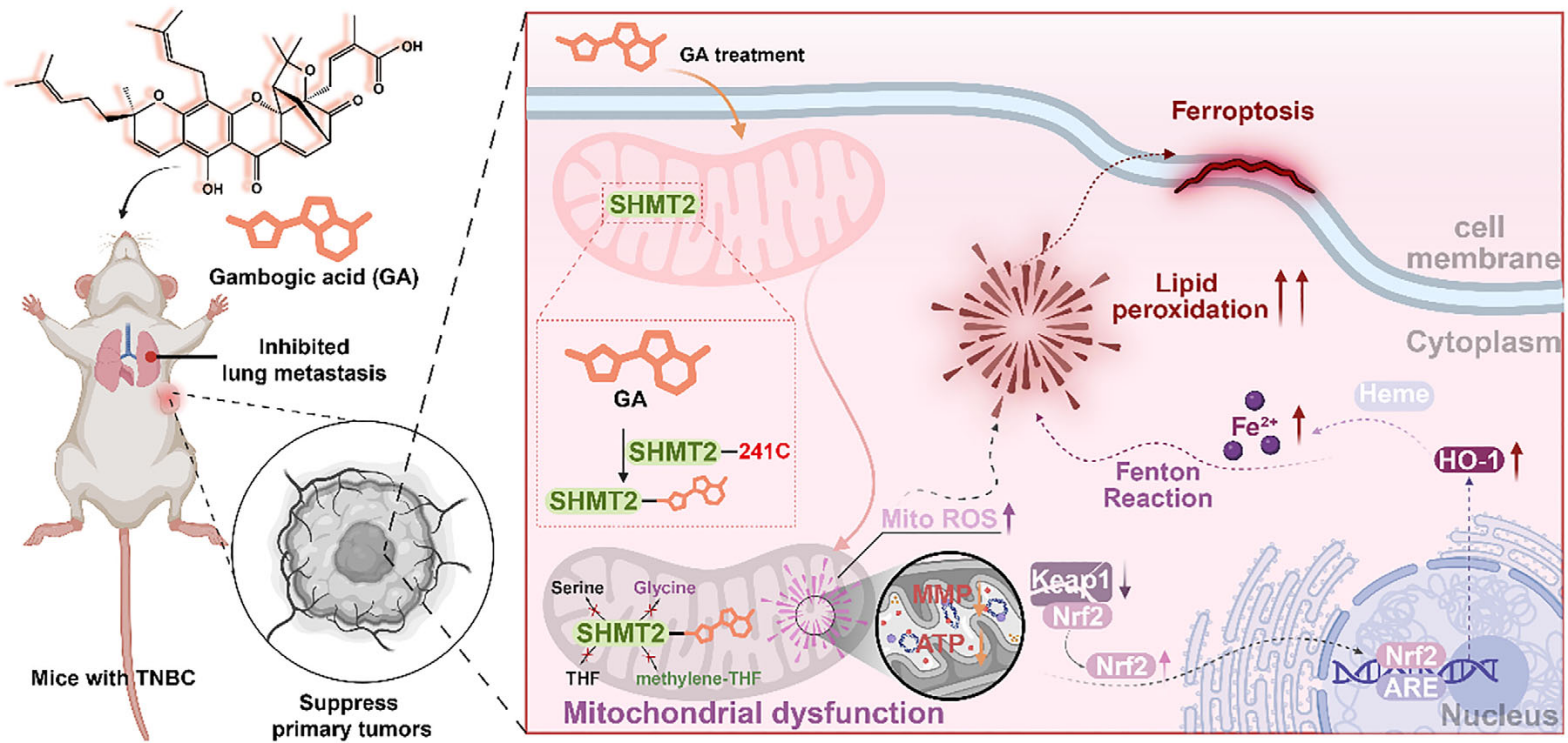
Schematic illustration of GA‐mediated inhibition of TNBC via targeting SHMT2. GA covalently binds to and inhibits SHMT2, thereby disrupting one‐carbon metabolism and mitochondrial homeostasis. This inhibition leads to mitochondrial dysfunction, activation of the Nrf2/HO‐1 signaling axis, and induction of iron overload and lipid peroxidation, ultimately triggering ferroptosis and suppressing TNBC progression.

TNBC presents a significant clinical obstacle due to its aggressive nature, strong tendency to metastasize, and, most critically, the absence of effective targeted treatments stemming from a lack of canonical molecular drivers [1, 2]. This therapeutic void underscores the urgent need to identify and exploit TNBC‐specific vulnerabilities. In this study, we demonstrated that GA, a natural compound with known anticancer activity but previously untested in TNBC–effectively suppresses TNBC progression in both in vitro and in vivo experimental models. By utilizing a combination of chemical proteomic techniques and functional analyses, we identified SHMT2 as the principal covalent target of GA in TNBC cells. GA covalently modifies the Cys241 residue of SHMT2, thereby inhibiting its enzymatic activity and disrupting its oligomerization. Importantly, we delineate a novel mechanistic cascade initiated by SHMT2 inhibition, which induces catastrophic mitochondrial dysfunction, manifested by bioenergetic failure, antioxidant depletion, and structural degradation. This mitochondrial collapse activates the Nrf2/HO‐1 signaling pathway, culminating in iron overload, excessive lipid peroxidation, and ultimately ferroptosis. Additionally, we establish that SHMT2 overexpression is a hallmark of TNBC, correlating with poor clinical prognosis, and that SHMT2 plays an indispensable oncogenic role by preserving mitochondrial integrity, thereby suppressing ferroptosis and promoting tumor progression (Figure 10). Collectively, our findings deliver significant scientific and translational insights: (i) we identified SHMT2 as a novel, druggable vulnerability in TNBC and position GA as the first‐in‐class covalent inhibitor targeting this enzyme; (ii) we revealed an unprecedented SHMT2‐mitochondria‐Nrf2/HO‐1‐ferroptosis regulatory axis, substantially advancing our understanding of TNBC biology and regulated cell death mechanisms; and (iii) we provided a compelling preclinical rationale and mechanistic foundation for repurposing GA or developing novel SHMT2‐targeted ferroptosis inducers as innovative therapeutic strategies for this therapeutically refractory cancer.

Importantly, we identified SHMT2 as a direct covalent target of GA in TNBC cells. GA inactivates SHMT2 by covalently modifying the cysteine residue at position 241, a previously unreported mechanism. Notably, the nearby lysine residue (Lys280), which forms a Schiff base with the cofactor PLP, is critical for SHMT2's catalytic activity [19]. Our results indicate that dysregulated SHMT2 expression is a critical driver of TNBC progression. SHMT2 facilitates mitochondrial one‐carbon folate metabolism, thereby supporting purine and thymidine biosynthesis essential for cell proliferation while maintaining mitochondrial integrity and metabolic homeostasis. Moreover, SHMT2 loss impairs mitochondrial translation, disrupts the synthesis of respiratory chain proteins, and causes defects in oxidative phosphorylation, all hallmark features of mitochondrial dysfunction [38, 39]. Thus, strategies aimed at SHMT2 degradation or comprehensive inhibition may represent effective approaches to suppress tumor growth. Consistent with this, our study shows that GA exerts markedly greater cytotoxic effects on TNBC cells compared to non‐TNBC cells, primarily by disrupting mitochondrial homeostasis. Specifically, GA treatment significantly reduces the NAD^+^/NADH ratio, inhibits ATP production, and depletes GSH levels, which in turn enhances the sensitivity of TNBC cells to oxidative stress and ferroptosis. Additionally, these results underscore the potential of SHMT2‐mediated mitochondrial metabolism as a valuable therapeutic target for cancers that are resistant to treatment.

Nuclear factor erythroid 2‐related factor 2 (Nrf2) plays a crucial role in regulating cellular defense mechanisms, facilitating adaptation to both oxidative and electrophilic stress [40, 41] and is a key therapeutic target [42]. The signaling pathway involving Nrf2 and HO‑1, known for its cytoprotective properties, can exert context‑dependent, dual effects on ferroptosis [43]. Activation of Nrf2 upregulates antioxidant genes, including HO‑1, conferring protection against ROS, HO‐1‐mediated heme degradation concurrently releases free Fe^2+^, which could exacerbate lipid peroxidation [44, 45]. Within the tumor microenvironment, this duality creates a paradox where the Nrf2/HO‐1 axis may either promote tumor survival by counteracting oxidative stress or trigger ferroptosis through iron‐mediated lipid peroxidation under specific conditions [46]. This “double‐edged sword” effect remains poorly understood in TNBC, especially in relation to mitochondrial damage and its regulatory mechanisms. Our mechanistic investigations reveal that SHMT2 inhibition‐induced mitochondrial dysfunction activates the Nrf2/HO‐1 pathway. Although Nrf2 activation is generally cytoprotective, the resultant rapid and intense upregulation of HO‐1 promotes heme degradation and releases free Fe^2+^, which, combined with mitochondrial redox imbalance, intensifies lipid peroxidation and induces ferroptosis [45, 47, 48]. GA‐triggered SHMT2 inhibition converts HO‐1 from a cytoprotective factor into a ferroptosis executor. This mechanism aligns with emerging evidence that excessive HO‐1‐driven iron accumulation synergistically enhances ferroptosis in cancer [45]. Given TNBC's notorious resistance to apoptosis‐inducing therapies, our findings highlight GA's ability to circumvent this resistance by inducing ferroptosis via a cascade involving mitochondrial collapse, Nrf2/HO‐1 activation, iron overload, and lipid peroxidation. This non‑apoptotic mode of cell death represents a potential therapeutic approach to surmount the inherent chemoresistance of TNBC.

GA has been extensively documented to induce apoptosis across diverse tumor cell types [49], and our findings confirm that GA treatment significantly triggers apoptosis in TNBC cells, accompanied by pronounced ferroptotic features. Most critically, time‐course analysis using specific inhibitors DFO and Z‐VAD‐FMK at 6‐ and 24‐h time points confirmed that while cellular injury may trigger secondary apoptotic features, ferroptosis represents the primary upstream driver of GA‐induced cell death (Figure S12). We reasonably hypothesize that GA‐mediated inhibition of SHMT2 underlies this dual mode of cell death in a time‐dependent manner. By targeting SHMT2, GA may impair mitochondrial one‐carbon metabolism and redox homeostasis, thereby promoting intracellular iron accumulation, lipid peroxidation, and ROS generation. The resulting oxidative damage to membranes and mitochondria is likely to activate intrinsic apoptotic signaling, ultimately culminating in TNBC cell apoptosis. While SHMT2 inhibition appears to be the primary mechanism underlying GA's potent anti‐TNBC activity, our chemoproteomic profiling further suggests that the anti‐TNBC activity of GA is unlikely to depend on SHMT2 alone, but rather reflects a broader multi‐target pharmacological mechanism. In addition to SHMT2, GA has also been reported to covalently target several functionally important proteins, including members of the HSP and USP protein families, as well as 6PGD [27, 30, 31]. These targets may not act independently, but instead could participate in an interconnected stress network that converges on metabolic collapse, impaired proteostasis, and loss of adaptive survival capacity in TNBC cells. Among them, the concomitant targeting of SHMT2 and 6PGD is particularly noteworthy. SHMT2 supports mitochondrial one‐carbon flux and cellular redox homeostasis, whereas 6PGD is a key enzyme in the pentose phosphate pathway (PPP) that contributes to NADPH production. Therefore, simultaneous inhibition of these two metabolic nodes could, in principle, impose a dual blockade on antioxidant buffering, reduce the capacity of TNBC cells to detoxify lipid peroxides, and lower the threshold for ferroptosis execution. In this sense, our data are consistent with a potential synthetic lethal‐like interaction at the level of redox homeostasis, although this possibility will require direct validation by combinatorial genetic or pharmacological perturbation. At the same time, the reported involvement of HSP‐ and USP‐family proteins expands the interpretation of GA action beyond metabolic regulation alone. HSP‐family proteins are central molecular chaperones required for proteome stability under stress conditions, whereas USP‐family proteins contribute to protein deubiquitination and maintenance of signaling protein turnover. Interference with these protein families may compromise the ability of TNBC cells to buffer proteotoxic stress and to engage compensatory survival pathways in response to metabolic injury. Thus, while SHMT2 may act as a primary mechanistic hub linking GA to ferroptosis, additional engagement of HSP‐ and USP‐family proteins, together with 6PGD and other cysteine‐reactive targets, may further amplify cellular vulnerability by preventing adaptive recovery. This integrated view may also help explain why GA produces such potent cytotoxicity in aggressive TNBC cells, which are highly dependent on metabolic plasticity and effective protein quality control for survival. Future studies incorporating combinatorial gene silencing, rescue experiments, and integrated multi‐omics analyses will be necessary to determine whether simultaneous perturbation of SHMT2 with 6PGD or other GA‐sensitive targets generates synthetic lethal interactions and to define how these target networks shape the therapeutic window of GA in TNBC. For clinical translation, a thorough evaluation of GA's safety profile is essential. Although GA demonstrates efficacy with manageable acute toxicity in vivo at 2–6 mg/kg, the long‐term consequences of sustained HO‐1 activation, implicated in inflammation, iron homeostasis, and possible pro‐tumorigenic effects, remain unclear. Moreover, on‐target toxicities, particularly bone marrow suppression resulting from SHMT2's role in nucleotide synthesis in hematopoietic stem cells, represent potential risks not captured in short‐term models. Comprehensive toxicological assessments across multiple species and extended dosing regimens, together with detailed pharmacokinetic characterization, are critical. Addressing current limitations such as poor solubility, metabolic instability, and undefined parameters, including bioavailability, tissue penetration, and elimination half‐life, will be necessary to advance GA toward clinical application. Future strategies should prioritize optimization of GA's pharmaceutical profile through innovative formulations, prodrug design, or development of derivatives with improved selectivity and ADME properties, thereby maximizing its therapeutic potential against TNBC (Figure 10).

## 4 Conclusions

In summary, our study identified SHMT2 as a critical metabolic vulnerability in TNBC and establishes GA as a covalent inhibitor targeting this enzyme. We reveal that GA selectively binds to the functionally essential cysteine residue Cys241 of SHMT2, disrupting its enzymatic activity and oligomerization, which leads to catastrophic mitochondrial dysfunction. This dysfunction activates the Nrf2/HO‑1 antioxidant signaling cascade, leading to excess intracellular iron, enhanced lipid peroxidation, and the induction of ferroptosis. Importantly, we demonstrate that SHMT2 is highly overexpressed in TNBC and is indispensable for maintaining mitochondrial integrity and suppressing ferroptosis, thereby promoting tumor progression. Our integrated chemical proteomic, biochemical, and functional analyses illuminate a novel SHMT2‐mitochondria‐Nrf2/HO‐1‐ferroptosis regulatory axis that underpins GA's potent anti‐TNBC efficacy. This work enhances the understanding of the mechanisms behind TNBC metabolism and the regulation of ferroptosis, while also underscoring the potential of SHMT2 as a valuable target for therapy. The discovery of GA's covalent inhibition of SHMT2 provides a compelling foundation for developing innovative treatments aimed at exploiting ferroptosis induction to overcome the clinical challenges posed by aggressive TNBC.

## 5 Experimental Section

### 5.1 Reagents and Materials

Gambogic acid (GA, with a purity of ≥ 98%) was sourced from Beijing Bethel Human Biomedical Technology Co., Ltd. in Beijing, China. For the click‐chemistry, pull‐down assays, and LC‐MS/MS, reagents such as CuSO_4_, TAMRA‐azide, Tris(2‐carboxyethyl) phosphine hydrochloride (TCEP), and Tris[(1‐benzyl‐1H‐1,2,3‐triazol‐4‐yl) methyl] amine (TBTA) were acquired from Sigma–Aldrich in the USA. Additionally, high‐capacity NeutrAvidin agarose beads, tetraethylammonium bromide (TEAB), sequencing‐grade modified trypsin, LC‐MS‐grade dithiothreitol and iodoacetamide, the TMT10plex reagent kit, and the Pierce Quantitative Fluorometric Peptide Assay Kit were purchased from Thermo Fisher Scientific located in the USA. Primary antibodies targeting SHMT2 (11099‐1‐AP), Keap1 (60027‐1‐IG), Nrf2 (16396‐1‐AP), HO‐1 (66743‐1‐IG), NQO1 (67240‐1‐IG), and β‐actin (66009‐1‐IG) were obtained from Proteintech in Wuhan, China.

### 5.2 Cell Culture

MDA‐MB‐231 (CVCL_0062), Hs578T (CVCL_0332), 4T1 (CVCL_0125), MCF‐7 (RRID: CVCL_0031), T47D (RRID: CVCL_0553), MCF‐10A (RRID: CVCL_0598), SK‐MES‐1 (RRID: CVCL_0630), HepG2 (RRID: CVCL_0027), HT1080 (RRID: CVCL_0317), and B16 (RRID: CVCL_F936) cells were obtained from the Chinese Academy of Medical Sciences, located in Beijing, China. All the cell lines were confirmed to be mycoplasma‐free. The cultures were kept in either high‐glucose Dulbecco's Modified Eagle Medium (DMEM; Gibco, USA) or Roswell Park Memorial Institute‐1640 medium (RPMI‐1640; Gibco, USA), both enriched with 10% fetal bovine serum (FBS; Corning, USA) and 1% penicillin–streptomycin (Gibco, USA), and were incubated at 37°C in a humidified environment with 5% CO_2_.

### 5.3 Cell Viability Assay

The evaluation of in vitro proliferation was carried out using the Cell Counting Kit‑8 (MA0225, Meilunbio) in accordance with the instructions provided by the manufacturer. Proliferation curves were generated from standardized optical density measurements at 450 nm (OD_450_), and cell survival was expressed as the percentage change in absorbance before and after each treatment.

### 5.4 Lentiviral shRNA Knockdown

MDA‐MB‐231 and Hs578T cell lines were modified to achieve stable knockdown of SHMT2 through lentiviral transduction. Lentiviral particles were generated by co‐transfecting HEK293T cells with shSHMT2 constructs, psPAX2, and pMD2.G (Invitrogen, CA, USA) using Lipofectamine 2000, followed by 48 h incubation. Viral supernatants were harvested, filtered through a 0.45 µm membrane, and stored at −80°C. TNBC cells (1 × 10^4^ cells per well) seeded in 6‐cm dishes were infected with viral solution for 48 h, and transduction efficiency was confirmed by fluorescence microscopy. Stable clones were selected with 4 µg/mL puromycin for one week, and SHMT2 knockdown was validated by Western blot. The shRNA target sequences were: shSHMT2‐1: 5′‐CTGACGTCAAGCGGATATC‐3′; shSHMT2‐2: 5′‐AAGTACTCGGAGGGTTATC‐3′.

### 5.5 Colony Formation Assay

TNBC cells were plated at a density of 1 × 10^3^ cells per well in six‐well plates, each of which contained 2 mL of complete culture medium. Following a 7‐day incubation period, the medium was discarded, and the cells were fixed using 4% paraformaldehyde (Servicebio; G1101‐500 mL, Wuhan, China) for a duration of 10 min. Subsequently, the cells were stained with 0.1% crystal violet (Solarbio, Beijing, China) for 30 min. The plates were then rinsed with tap water, allowed to air dry, photographed, and the colonies were quantified utilizing ImageJ software.

### 5.6 Cell Migration Assay

A confluent layer of cells in 6‑well plates was scraped in a linear manner using a 200 µL pipette tip to create a gap. Cells that had detached were eliminated through washing with PBS, and the cultures were then kept in complete medium, either with or without GA. Photographs of the wound area were captured at both 0 h and 24 h after scratching using an Olympus inverted microscope. The width of the gap was measured using ImageJ, with the initial measurement at 0 h set to 1, and the relative migration rate was determined based on the distance covered by the cells.

### 5.7 Cell Invasion Assay

Cell invasion assessment was conducted using 24‐well Transwell chambers that featured 8.0 µm polycarbonate membranes (14341, Labselect). The inserts' top surfaces were coated with 20 µL of Matrigel at a 1:2 dilution (ABWbio; 0827045), whereas the lower chambers were filled with 600 µL of medium enriched with 20% FBS. TNBC cells treated with GA (5 × 10^4^ per well; 0–1.20 µm) were resuspended in 100 µL of basal medium and placed into the upper chamber. After 24 h of incubation, membranes were stained with 0.1% crystal violet, and invading cells were quantified under an Olympus inverted microscope.

### 5.8 Live/Dead Cell Staining of 3D Multicellular Tumor Spheroids

A 0.75% agarose solution was prepared in PBS, sterilized by autoclaving, and dispensed while hot into 96‑well plates (60 µL/well). The plates were exposed to UV light for 30 min and cooled to solidify. Each well was overlaid with a TNBC cell suspension containing 1 × 10^3^ cells. MCTS (multicellular tumor spheroids) developed over a span of 2 to 3 days and were preserved at 37°C prior to the administration of GA (ranging from 0 to 1.2 µm) for a duration of 24 h. After the treatment, the spheroids were rinsed with PBS, treated with calcein‐AM and propidium iodide (PI) for 30 min, and examined using a confocal laser scanning microscope (CLSM; Andor).

### 5.9 Apoptosis Assay

Triple‐negative breast cancer (TNBC) cells were plated at a density of 3.5 × 10^5^ cells per well in six‐well plates, incubated overnight, and then exposed to GA (0–1.2 µm) for 24 h. After the treatment period, cells were collected using trypsin without EDTA (Gibco, USA) and rinsed three times with PBS. Annexin V‐FITC staining (Beyotime, C1062M) was carried out for 20 min, and subsequently, apoptosis was assessed using flow cytometry (Beckman Coulter).

### 5.10 Animal Experiments

Five‐week‐old female BALB/c or nude mice were acquired from Vital River Experimental Animal Technology Co., Ltd. (Beijing, China). Either 4T1 cells (5 × 10^5^) or MDA‐MB‐231 cells with SHMT2 knockdown (2 × 10^6^) were injected orthotopically into the mammary fat pads. Upon tumor establishment, mice were randomly allocated to experimental groups at the initiation of treatment. For GA or PTX administration, injections were performed every two days using the following solutions: control saline, PTX (10 mg/kg), GA‐Low (2 mg/kg), and GA‐High (6 mg/kg). For the lung metastasis model, after in situ tumors were established, tumor‐bearing mice were injected intravenously with 5 × 10^5^ 4T1 cells. On day 15, lung tissue was excised and examined under bright light to count nodules. Following fixation in 4% paraformaldehyde, the number of nodules was further evaluated by hematoxylin and eosin (HE) staining. In SHMT2 knockdown assays, mice received injections of shNC, shSHMT2‐1, or shSHMT2‐2 cells. Tumor dimensions were recorded every two days with a Vernier caliper, and volumes were calculated using the formula: (length × width^2^) × 0.5. Mice were euthanized when total tumor volume exceeded 1,500 mm^3^. All animal studies complied with institutional animal welfare regulations and received approval from the Animal Ethics and Welfare Committee at Shenzhen People's Hospital (AUP‑250217‑ZY‑0087‑01).

### 5.11 Competitive in‐Gel Fluorescent Labeling of GA in TNBC Cells

Cells of TNBC (2 × 10^6^ per well) were plated in 6 cm dishes and allowed to grow until reaching 80%–90% confluence. Following this, the cells were subjected to treatment with GA at concentrations of 0, 0.1, 0.3, 0.6, and 1.2 µm for a duration of 12 h. After treatment, the cells were rinsed three times with PBS, harvested by scraping, and centrifuged at 500 × g for 5 min at 4°C, discarding the supernatant. The resulting pellets were then resuspended in 100 µL of lysis buffer that included 1% protease inhibitor (Meilunbio, MB2678) along with 0.2% Triton X‐100 (Beyotime, ST1723‐100ML) and sonicated on ice at an amplitude of 20% until the solution became clear. The lysates were clarified through centrifugation at 20,000 × g for 20 min at a temperature of 4°C and subsequently diluted to a protein concentration of 2 mg/mL using PBS. For each experimental group, 200 µg of protein was incubated alongside the cysteine‐specific probe IAA‐P at 37°C while being shaken at 1,200 rpm for 1 h. The click chemistry process commenced with the addition of 16 µL of click reaction buffer, 9 µL of TBTA (10 mm in DMSO), 3 µL of TCEP (50 mm in H_2_O), 3 µL of CuSO_4_ (50 mm in H2O), and 1 µL of TAMRA‐azide (10 mm in DMSO), followed by a 2‐h incubation at 37°C with shaking. To precipitate the proteins, 1 mL of cold acetone was added, and the mixture was incubated at −20°C for 30 min before pelleting at 20,000 × g for 10 min at 4°C. Pellets were resuspended in 50 µL 1× SDS loading buffer (Beyotime, P0015), denatured at 95°C for 10 min, and separated on 12% SDS‐PAGE. Fluorescent gels were imaged with an Azure Sapphire RGB‐NIR scanner (Azure Biosystems, USA), and total protein was visualized by Coomassie Brilliant Blue staining (Beyotime, P0017).

In competitive labeling assays using recombinant proteins (1 µg/µL), the samples were incubated with the competitor (GA or IAA) for 1 h, followed by a reaction with 5 µm IAA‐P for another hour at room temperature while agitating. Afterward, the reaction mixture was subjected to a 1‐h click chemistry step utilizing a pre‐prepared buffer. Ultimately, the proteins were denatured in SDS loading buffer, separated by SDS‐PAGE, and detected according to the methods previously outlined.

### 5.12 Cysteine‐Based Protein Profiling Workflow

To identify potential protein targets of GA in TNBC, a competitive cysteine‐reactivity proteomics approach was utilized. In this procedure, protein lysates at a concentration of 200 µg were incubated with a DBIA probe at a concentration of 100 µm, sourced from Chomix Biotechnology in Nanjing, China, maintaining a temperature of 37°C for a duration of 1 h. Following the incubation, the labeled proteins underwent reduction using 10 mm DTT at the same temperature for 30 min, after which they were alkylated with 20 mm IAA in a dark environment for an additional 30 min. Post‐alkylation, the proteins were precipitated using 1 mL of ice‐cold acetone, and this mixture was subjected to centrifugation at 2000 × g for 20 min at 4°C. The resultant pellets were reconstituted in 100 µL of 100 mm TEAB, and a trypsin digestion step was performed at 37°C for 24 h using 10 µg of trypsin. After digestion, the peptides were labeled with TMT and then enriched using 160 µL of streptavidin beads in PBS at room temperature for a period of 24 h. This process was followed by a series of washes using PBS, a 0.1% SDS solution, and ultrapure water to ensure proper purification. Finally, the peptides underwent a desalting process on a C18 column utilizing a solution of 0.1% formic acid. The prepared samples were then subjected to LC‐MS/MS analysis, which allows for the subsequent identification and quantification of the proteins of interest in the context of TNBC.

### 5.13 Cellular Imaging

Fluorescence colocalization assays were conducted to investigate the interaction of GA with its potential target, SHMT2, in TNBC cells. The cells were plated in four‐well glass‐bottom confocal dishes (Cellvis), allowed to culture overnight, and treated with GA (at concentrations of 0 or 1.2 µm) for a duration of 12 h. After treatment, cells were incubated with 10 µm IAA‐P for 2 h, followed by three washes with 0.5 mL PBS. They were fixed using 4% paraformaldehyde for 10 min and then permeabilized with 0.2% Triton X‐100 for 15 min. A copper‐catalyzed azide–alkyne cycloaddition (“click” reaction) was performed at room temperature for 2 h. This was followed by overnight immunostaining with anti‐SHMT2 antibody (dilution 1:500) at 4°C and application of Alexa Fluor 488‐conjugated secondary antibody (dilution 1:500) for 2 h. Finally, nuclei were stained with Hoechst 33342 (dilution 1:5000) for 20 min, and fluorescence images were acquired using a CLSM.

### 5.14 In Situ Pull‐Down Experiment

To confirm the direct interaction between SHMT2 and GA, a streptavidin pull‐down assay was conducted under three different conditions: a blank control, application of the DBIA probe alone, and GA‐competitive labeling with DBIA. After precipitating proteins with acetone, they were resuspended in 1.2% SDS in PBS (1 mL) and denatured by heating at 90°C for 10 min. The samples were subsequently diluted to 0.2% SDS using PBS (5 mL) and incubated with 80 µL of streptavidin magnetic beads at room temperature for 4 h. Following centrifugation at 1,400 × g for 3 min to eliminate unbound proteins, the proteins attached to the beads were washed sequentially with 1% SDS/PBS, 0.1% SDS/PBS, and 6 M urea/PBS. The target proteins were then eluted in 1× SDS loading buffer by heating at 95°C for 10 min, separated by SDS‐PAGE, and examined via Western blot with an anti‐SHMT2 antibody.

### 5.15 Cellular Thermal Shift Assay (CETSA)

To perform the cellular thermal shift assay, soluble proteins were extracted from MDA‐MB‐231 cells utilizing the lysis buffer described earlier. Equal amounts of protein were incubated with either GA or DMSO at a temperature of 37 °C, followed by exposure to a thermal gradient ranging from 37 °C to 65 °C in 4 °C increments (a total of eight temperature points). After heating, the samples underwent centrifugation at 2000 × g for 20 min, and the supernatants obtained were combined in equal volumes with SDS‐PAGE loading buffer for further Western blot analysis.

### 5.16 Expression and Purification of Recombinant Human SHMT2

The process of purifying recombinant proteins is akin to the method previously outlined [50], albeit with minor adjustments. The detailed process is as follows: The human SHMT2 protein, along with its mutants, underwent subcloning into the pET28a vector, allowing for the incorporation of an N‐terminal 6×His tag (Genereybiotech, Beijing, China). Plasmids were transformed into Escherichia coli BL21 cells, which were cultured in Luria‐Bertani (LB) medium containing 50 µg/mL kanamycin at 37°C and 200 rpm until the OD_600_ reached 0.6‐0.8. Protein expression was induced with 0.4 mm IPTG at 16°C for 18 h. Cells were collected by centrifugation, resuspended in lysis buffer (50 mm Tris‐HCl, pH 7.6; 300 mm NaCl; 20 mm imidazole; 1 mm DTT; 5% glycerol; 0.8 mm PMSF), and lysed by sonication. The lysate was clarified through centrifugation (20 000 × g for 20 min at 4°C), followed by loading the supernatant onto a Ni‐NTA agarose column (Qiagen, USA). Subsequent to washing with 50 mL of wash buffer (composed of 50 mm Tris‐HCl, pH 7.6; 300 mm NaCl; 20 mm imidazole; and 1 mm DTT), the proteins that bound were eluted using the same buffer that contained imidazole at concentrations ranging from 50 to 500 mm. The concentrations of the proteins were measured using a BCA assay, and their purity was verified via Coomassie‐stained SDS‐PAGE. The purified proteins were then concentrated and stored in a buffer solution (20 mm HEPES, pH 7.5; 200 mm NaCl; 5 mm DTT; and 10% glycerol).

### 5.17 SHMT2 Enzyme Activity Detection

SHMT2 catalyzes the conversion of DL‐β‐phenylserine to benzaldehyde, which exhibits maximal absorbance at 279 nm. Assays were performed at 25°C in buffer containing 100 µm potassium phosphate, 50 µm pyridoxal 5′‐phosphate (PLP), 10 µm tetrahydrofolate, and 5 µm β‐mercaptoethanol. Equal aliquots of SHMT2 were preincubated with varying concentrations of GA before initiating the reaction with 50 mm DL‐β‐phenylalanine. Following swift mixing, alterations in absorbance at 279 nm were measured using a multimode microplate reader to quantify the production of benzaldehyde, and the EC_50_values were determined from the corresponding dose‐response curves.

### 5.18 Determination of SHMT2‐GA Binding Affinity by Microscale Thermophoresis (MST) Assay

The dissociation constant of equilibrium (K_D_) for the binding of GA to recombinant human SHMT2, both wild‐type and mutant forms, was measured employing a Monolith NT.115 device (NanoTemper Technologies), following the methodologies detailed in prior studies [50]. The analysis of the data was conducted using MO. Affinity Analysis software, version 2.3.

### 5.19 Circular Dichroism (CD) Spectroscopy Analysis

Circular dichroism (CD) spectroscopy was performed on a JASCO J‐815 spectrometer (JASCO, Japan) to assess structural alterations in SHMT2. CD spectra of 20 µm SHMT2 were recorded at 298 K, with or without GA, using a 1 mm path‐length quartz cuvette. Spectra were collected in the range of 190 to 260 nm with a scanning rate of 50 nm/min; each spectrum reflects the average of three successive scans. The composition of secondary structures (including helices, β‐turns, parallel, antiparallel, and random coils) was analyzed using the CDNN software (version 2.1).

### 5.20 SHMT2 Cross‐Linking Assay

Protein oligomerization in the samples was analyzed via cross‐linking assays using wild‐type and mutant recombinant human SHMT2. Equal quantities of SHMT2, pre‐incubated with different concentrations of GA, were combined with the reaction buffer and allowed to incubate for 15 min. Subsequently, 500 µL of 2 mm disuccinimidyl suberate (DSS; Aladdin, D155694) was introduced, and the reaction was carried out at room temperature for 30 min. The cross‐linking reaction was terminated by adding SDS‐PAGE loading buffer, followed by the separation of samples via SDS‐PAGE, with protein oligomers being visualized through Coomassie Brilliant Blue staining.

### 5.21 Mitochondrial Morphology and Function Analysis

The evaluation of mitochondrial membrane potential (MMP) in TNBC cells was conducted utilizing a JC‐1 assay kit (Beyotime, China). A total of 5×10^4 cells/mL were cultured in four‐well glass‐bottom confocal chambers, exposed to GA for a duration of 24 h, and subsequently stained with 10 µm JC‐1 in the dark for 20 min. Following this, the cells were rinsed twice with PBS and visualized using CLSM. The intracellular levels of ATP, glutathione (GSH), and the ratios of NAD+/NADH were then quantified using specific detection kits (Beyotime, China). For mitochondrial visualization, the cells underwent two washes with warm, serum‐free medium and were treated with 250 nM PK Mito Red (Genvivo Biotech, PKMR‐1) along with Hoechst 33342 in 300 µL of serum‐free medium at 37°C for 15 min in the dark. They were washed again twice and imaged by CLSM to evaluate mitochondrial morphology. The examination of mitochondrial ultrastructure was carried out through transmission electron microscopy (TEM): GA‐treated cells were harvested, centrifuged, and fixed in 2.5% glutaraldehyde at 4°C for 4 h. Following dehydration, embedding, sectioning, and counterstaining, the samples were analyzed via TEM.

### 5.22 Seahorse Assay

The oxygen consumption rate (OCR) of TNBC cells was measured using the Seahorse extracellular flux analyzer (Seahorse Bioscience, USA). For this assay, GA‐treated TNBC cells were first cultured in Seahorse XFe 96‐well microplates. The OCR was then assessed using the mitochondrial stress assay kit, with subsequent procedures conducted according to the manufacturer's protocol. Data were processed using Seahorse XFe Wave software (Agilent Technologies, USA).

### 5.23 Measurement of Fe^2+^ in TNBC Cells

TNBC cells were seeded in six‐well plates. After 24 h of treatment, cells were incubated with 1 µm FerroOrange (Dojindo, M489) in the dark for 30 min and washed twice with PBS. Cells were detached using EDTA‐free trypsin, washed twice with PBS, resuspended in 300 µL PBS, and analyzed by flow cytometry for intracellular Fe^2+^ levels.

### 5.24 Detection and Quantification of Lipid Peroxidation

BODIPY 581/591 C11 (Thermo Scientific, #D3861) was employed to measure lipid peroxidation in cells subjected to various treatment conditions. After being treated for 24 h, TNBC cells were rinsed twice with PBS and then incubated at 37°C in the dark with medium containing 10 µm BODIPY 581/591 C11 for 30 min. Following this, the labeled cells were analyzed using CLSM and flow cytometry to evaluate lipid peroxidation levels.

### 5.25 Molecular Docking Analysis

Docking simulations were performed utilizing the Molecular Operating Environment (MOE; version 2019.01.02) with the default settings of the software. The conformer exhibiting the lowest docking score was identified as the potential binding mode. The chemical structure of GA was sourced from PubChem (CID: 9852185), while the SHMT2 crystal structure (PDB ID: 4PVF) was acquired from the Protein Data Bank and subsequently preprocessed through the MOE QuickPrep module. The highest‐ranking complex structure of SHMT2 with GA was then visualized using Pymol.

### 5.26 Proteomics and Data Analysis

Proteomic analysis was performed with modifications as previously outlined [51]. In summary, MDA‐MB‐231 cells were collected after being treated with or without GA, then lysed using RIPA buffer that included 1% protease inhibitor, and protein concentrations were quantified via the BCA assay. Proteins underwent reduction with 10 mm DTT and were subsequently alkylated for 30 min with 20 mm IAA. Following an overnight digestion using trypsin at 37°C, the resulting peptides were desalted on a C18 column. The samples were then examined through LC‐MS/MS with a Thermo Orbitrap Fusion Lumos (Thermo Scientific, USA), and the MS raw data were analyzed using Proteome Discoverer 2.4 (Thermo Scientific). Proteins exhibiting an absolute fold change ≥ 1.5 and a false discovery rate (FDR)‐adjusted *p*‐value < 0.05 were considered differentially expressed. Data visualization was performed using the BioLadder webserver (https://www.bioladder.cn) and TBtools. KEGG pathway enrichment and Gene Ontology analysis were performed via Cytoscape_v3.10.2 and the bioinformatics platform (http://www.bioinformatics.com.cn).

### 5.27 Statistical Analysis

Transcriptomic and clinical datasets for breast cancer were obtained from TCGA, GSE5460, and METABRIC. SHMT2 expression levels across breast cancer subtypes were quantified, and statistical significance was evaluated. Survival analyses stratified by SHMT2 expression were performed using GEPIA [52]. Statistical analyses were conducted in GraphPad Prism 9 (GraphPad, USA). Data were expressed as mean ± SEM. Multiple‑group comparisons were assessed by one‑way ANOVA followed by Tukey's post hoc test, and two‑group comparisons by two‑tailed Student's *t*‑test. A *p*‐value < 0.05 was considered statistically significant. Figure annotations: *p < 0.05; **p < 0.01; ***p < 0.001; ****p < 0.0001; ns, not significant.

## Author Contributions

Tong Yang, Chong Qiu, Yulei Li, and Ying Zhang designed the research, performed the majority of experiments, and drafted the manuscript. Jie Zhou, Chen Wang, Zheng Chu, and Junzhe Zhang performed the omics data analysis. Cui Liu, Peng Gao, Ang Ma, and Ling Huang helped conduct the animal experiments. Yin Kwan Wong helped to revise the manuscript. Junhua Zhang, Huan Tang, and Jigang Wang designed, supervised the research, and reviewed the manuscript.

## Conflicts of Interest

The authors declare no conflicts of interest.

## Supporting information




**Supporting file**: advs75011‐sup‐0001‐SuppMat.docx

## Data Availability

The data that support the findings of this study are available in the supplementary material of this article.
