# Supplementary material for: Covalent Inhibition of SHMT2 by Gambogic Acid Induces Ferroptosis Through Mitochondrial Collapse in Triple‐Negative Breast Cancer
Source: Adv Sci (Weinh). 2026 Jun 30;13(42):e20252. doi: 10.1002/advs.202520252 (PMC13325921; doi:10.1002/advs.202520252)
Supplement: Supplementary file 1 — Supporting File: advs75011‐sup‐0001‐SuppMat.docx. [file ADVS-13-e20252-s001.docx]

**Covalent Inhibition of SHMT2 by Gambogic Acid Induces Ferroptosis Through Mitochondrial Collapse in Triple-Negative Breast Cancer**

*Tong Yang^a,e,f,#^, Chong Qiu^b,#^, Yulei Li^c,#^, Ying Zhang^b,d,#^, Chen Wang^b^, Jie Zhou^b^, Zheng Chu^b^, Ang Ma^b^, Ling Huang^b^,Yin Kwan Wong^d^, Junzhe Zhang^b^, Peng Gao^b^, Cui Liu^b^,* *Junhua Zhang ^e,f,*^, Huan Tang^b,*^, Jigang Wang^a,b,e,f,*^*

a. School of Chinese Materia Medica, Tianjin University of Traditional Chinese Medicine, Tianjin 301617, China

b. State Key Laboratory for Quality Ensurance and Sustainable Use of Dao-di Herbs, Artemisinin Research Center, and Institute of Chinese Materia Medica, China Academy of Chinese Medical Sciences, Beijing 100700, China

c. Blood Transfusion Department, First Affiliated Hospital of Gannan Medical University, Key Laboratory of Prevention and Treatment of Cardiovascular and Cerebrovascular Diseases, Ministry of Education, Gannan Medical University, Ganzhou 341000, Jiangxi, China

d. Department of Pulmonary and Critical Care Medicine, Shenzhen Institute of Respiratory Diseases, Guangdong Provincial Clinical Research Center for Geriatrics, Shenzhen Clinical Research Center for Geriatrics, Shenzhen People's Hospital, The Second Clinical Medical College, Jinan University, Shenzhen 518020, Guangdong, China

e. State Key Laboratory of Chinese Medicine Modernization, Tianjin University of Traditional Chinese Medicine, Tianjin 301617, China

f. Haihe Laboratory of Modern Chinese Medicine, Tianjin 301617, China

^#^ These authors contributed equally to this work.

^*^ Corresponding authors.

E-mail addresses: [zjhtcm@foxmail.com](mailto:zjhtcm@foxmail.com) (Junhua Zhang), [htang@icmm.ac.cn](mailto:htang@icmm.ac.cn) (Huan Tang), [jgwang@icmm.ac.cn](mailto:jgwang@icmm.ac.cn) (Jigang Wang)


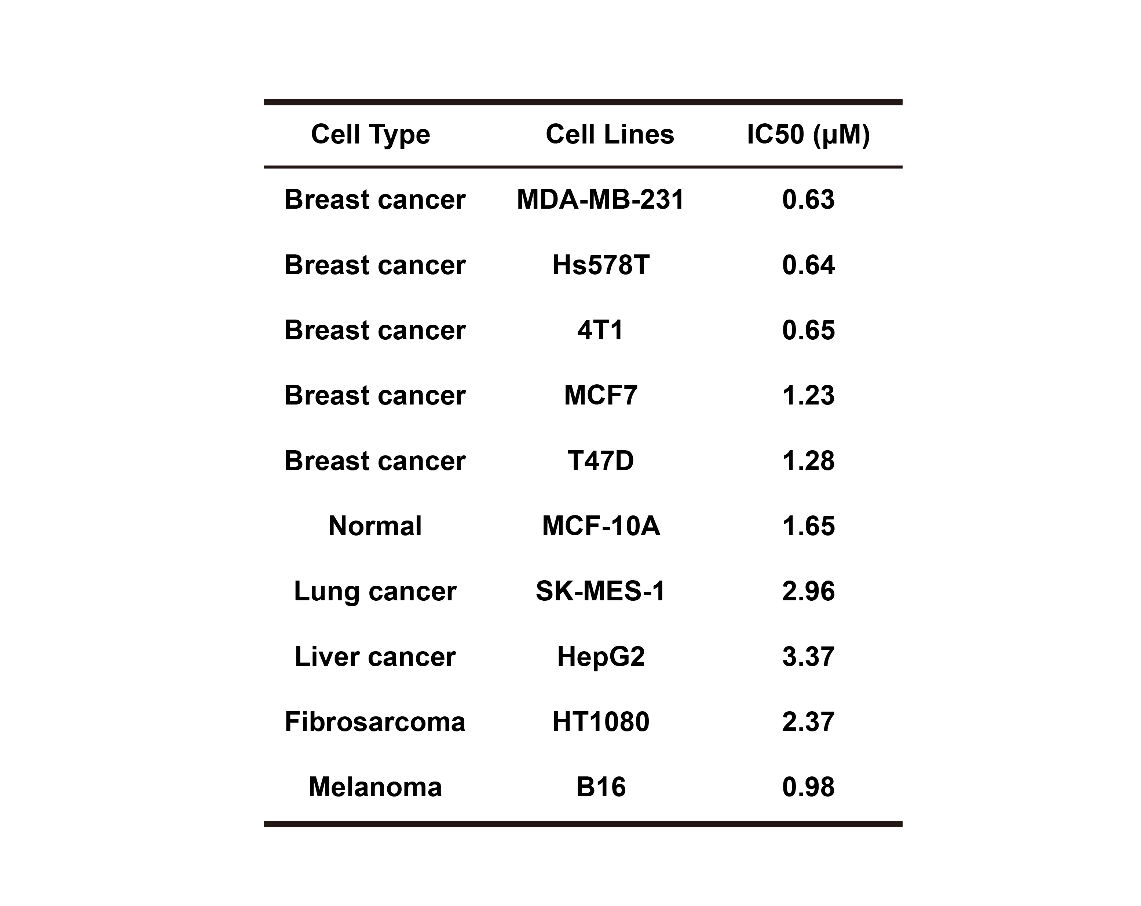


**Fig. S1.** IC_50_ of GA for various cell lines.


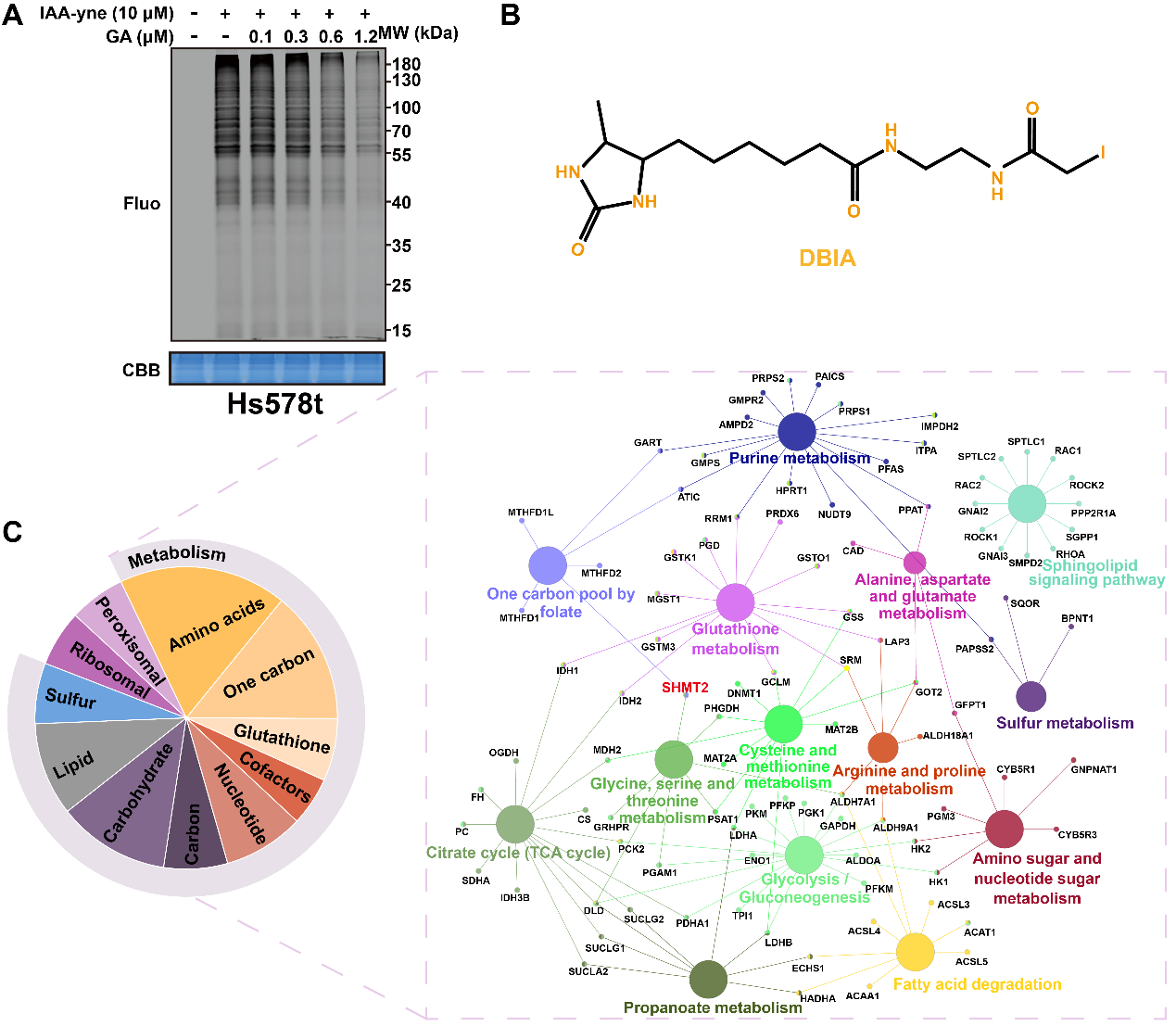


**Fig. S2.** (A) The competition of in situ protein labeling with IAA-yne by GA in TNBC cells. Fluo: Fluorescent image; CBB: Coomassie brilliant blue staining. (B) Chemical structure of DBIA. (C) Bioinformatic analysis of potential targets involved in metabolic processes in GA-sensitive proteins.


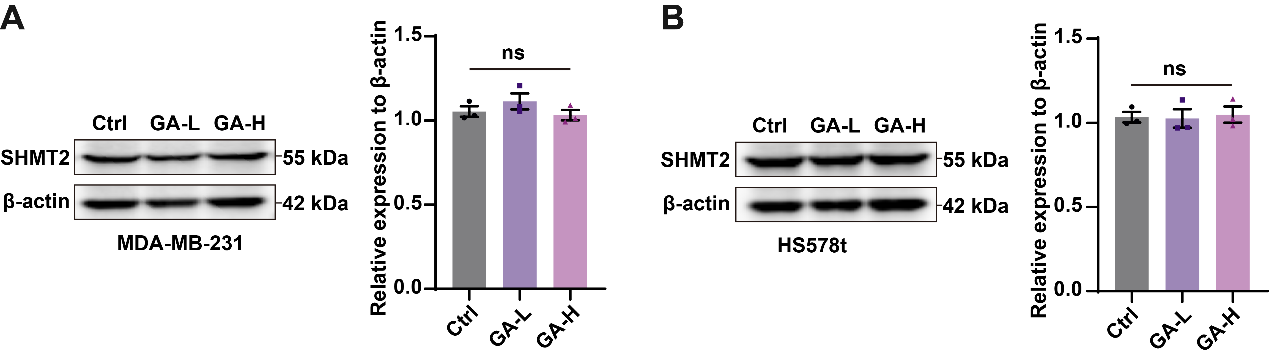


**Fig. S3.** (A-B) Treatment with GA did not affect the expression level of SHMT2 in TNBC cells.


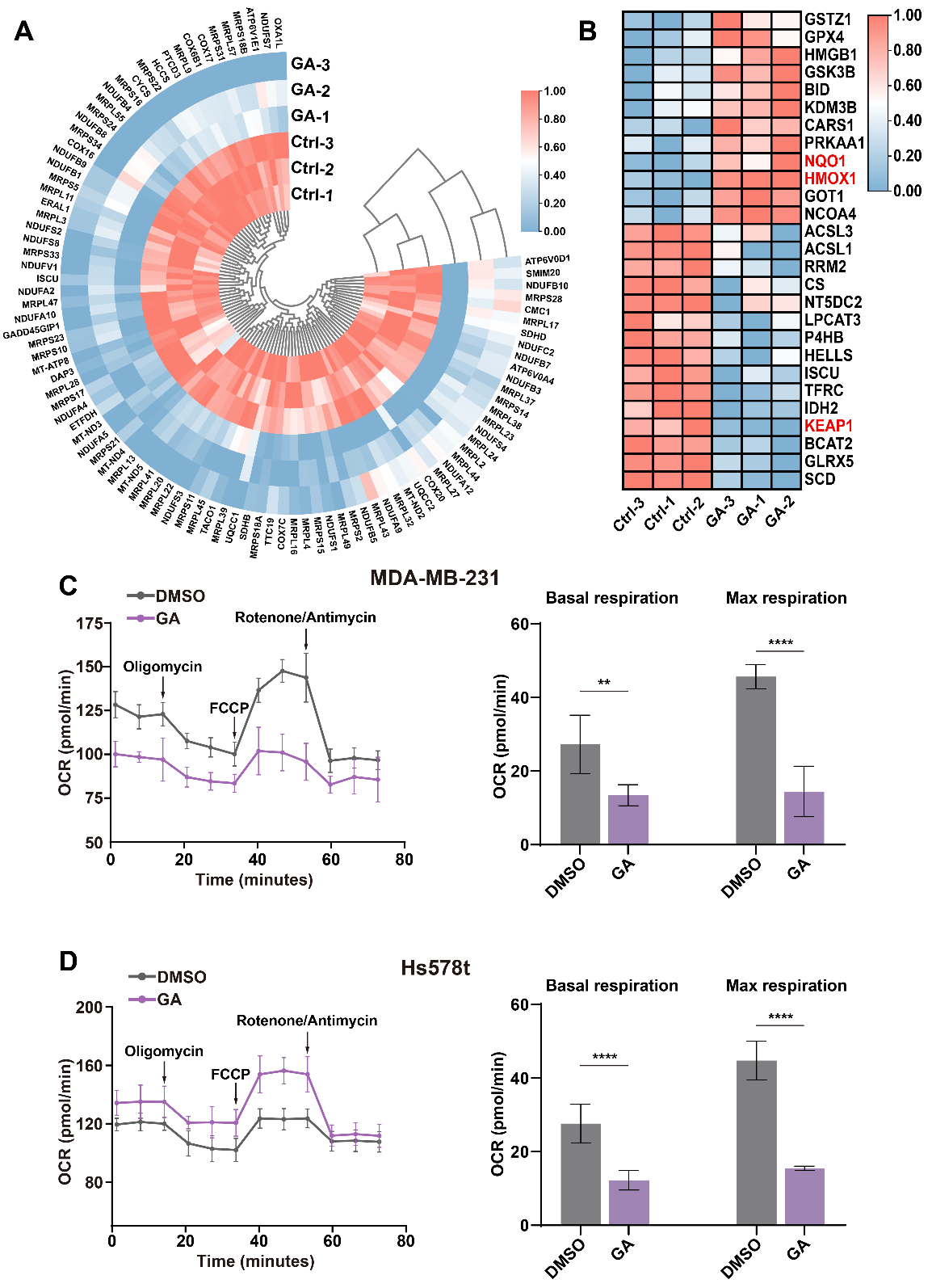


**Fig. S4.** (A) Effects of GA on the expression levels of oxidative phosphorylation-related proteins in TNBC cells. (B) Effect of GA on the expression levels of ferroptosis-related proteins in TNBC cells. (C) The OCR of MDA-MB-231 cells after GA and control DMSO treatment. The right panel presents statistical analysis of basal respiration and maximal respiration. (D) The OCR of Hs578T cells after GA and control DMSO treatment. The right panel presents statistical analysis of basal respiration and maximal respiration.


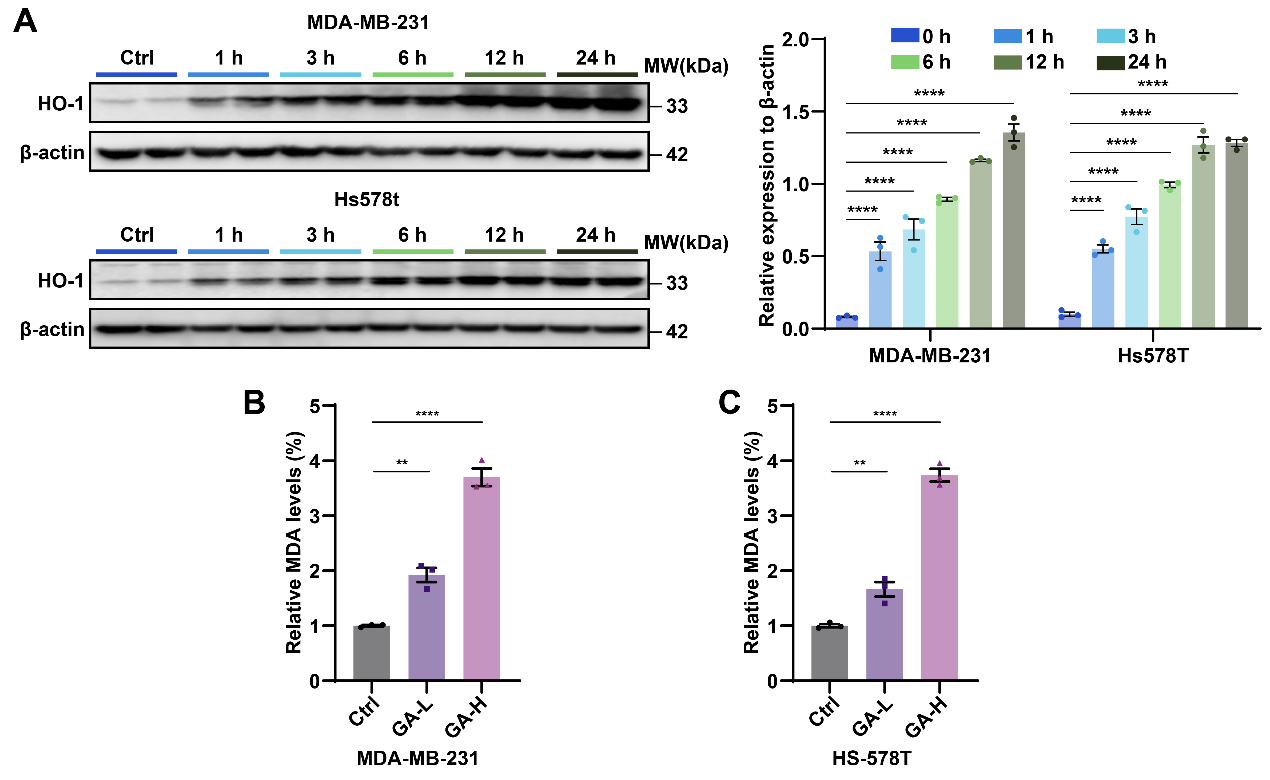


**Fig. S5.** (A) GA rapidly evoked substantial expression of HO-1 in TNBC. (B-C) GA significantly increased MDA levels in TNBC cells. Data were presented as means ± SEM (n = 3). Significance levels are indicated as ^*^p < 0.05, ^**^p < 0.01, ^***^p < 0.001, and ^****^p < 0.0001.


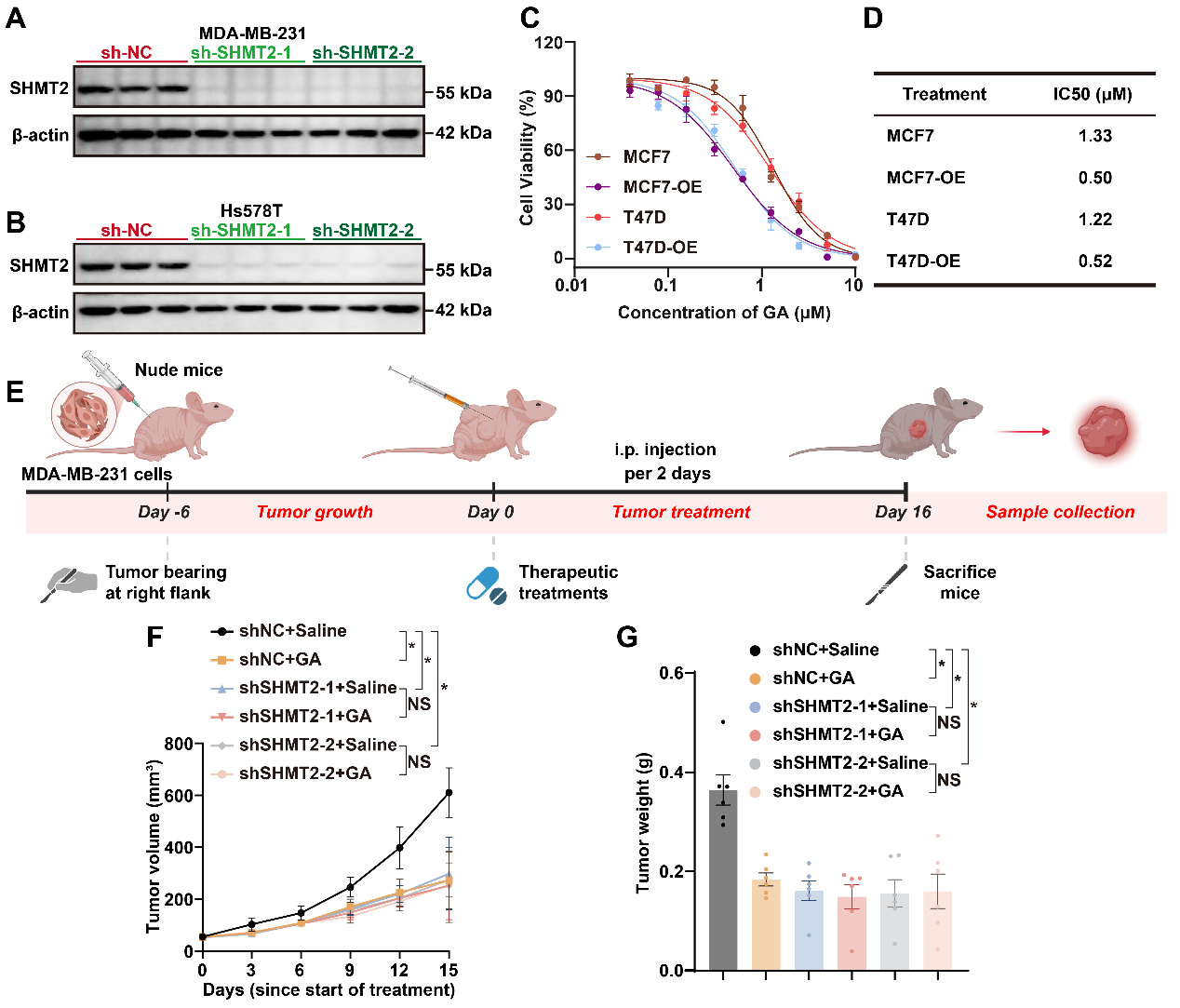


**Fig. S6.** (A-B) Establishment of SHMT2 knockdown TNBC cell lines. (C) Dose-response curves for cell viability following GA treatment in cell lines with low SHMT2 expression (MCF-7, T47D). (D) IC50 of GA for various cell lines. (E) Schematic illustration of the treatment regimen in MDA-MB-231 tumor bearing nude mice. (F-G) Examination on tumors formed by TNBC cells with SHMT2 knockdown: (F) Tumor volume from the mice with indicated treatment; (G) Weight of tumors from the mice with indicated treatment. Data were presented as means ± SEM (n = 6). Significance levels are indicated as ^*^p < 0.05.


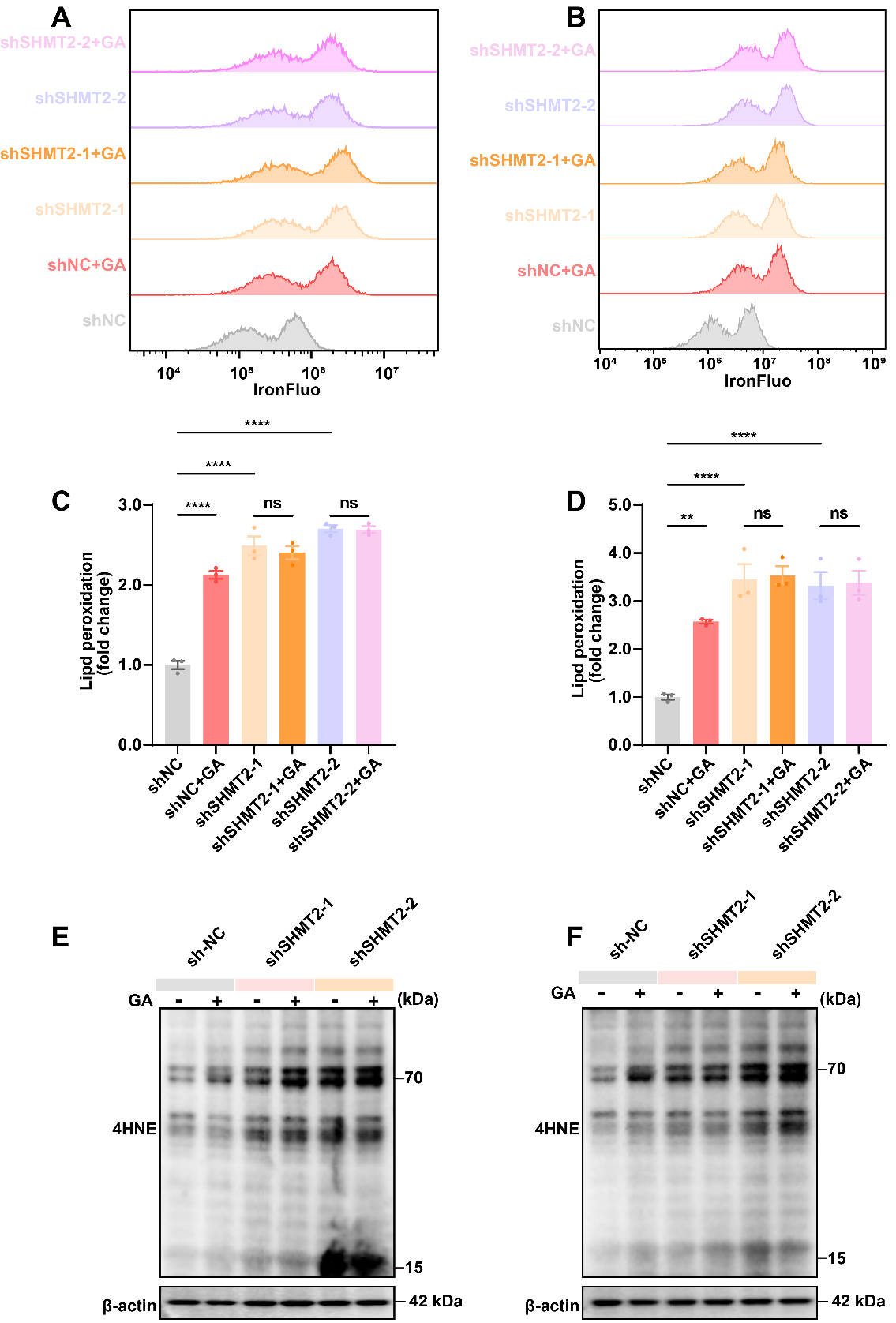


**Fig. S7.** (A-B) Fluorescence intensity distribution diagram for the statistical results in Fig. 8F–G. (C-D) Statistical results for the fluorescence distribution diagrams in Fig. 8H–I. (E-F) GA treatment significantly increased 4-HNE levels in TNBC cells. Data were presented as means ± SEM (n = 3). Significance levels are indicated as ^*^p < 0.05, ^**^p < 0.01, ^***^p < 0.001, and ^****^p < 0.0001.


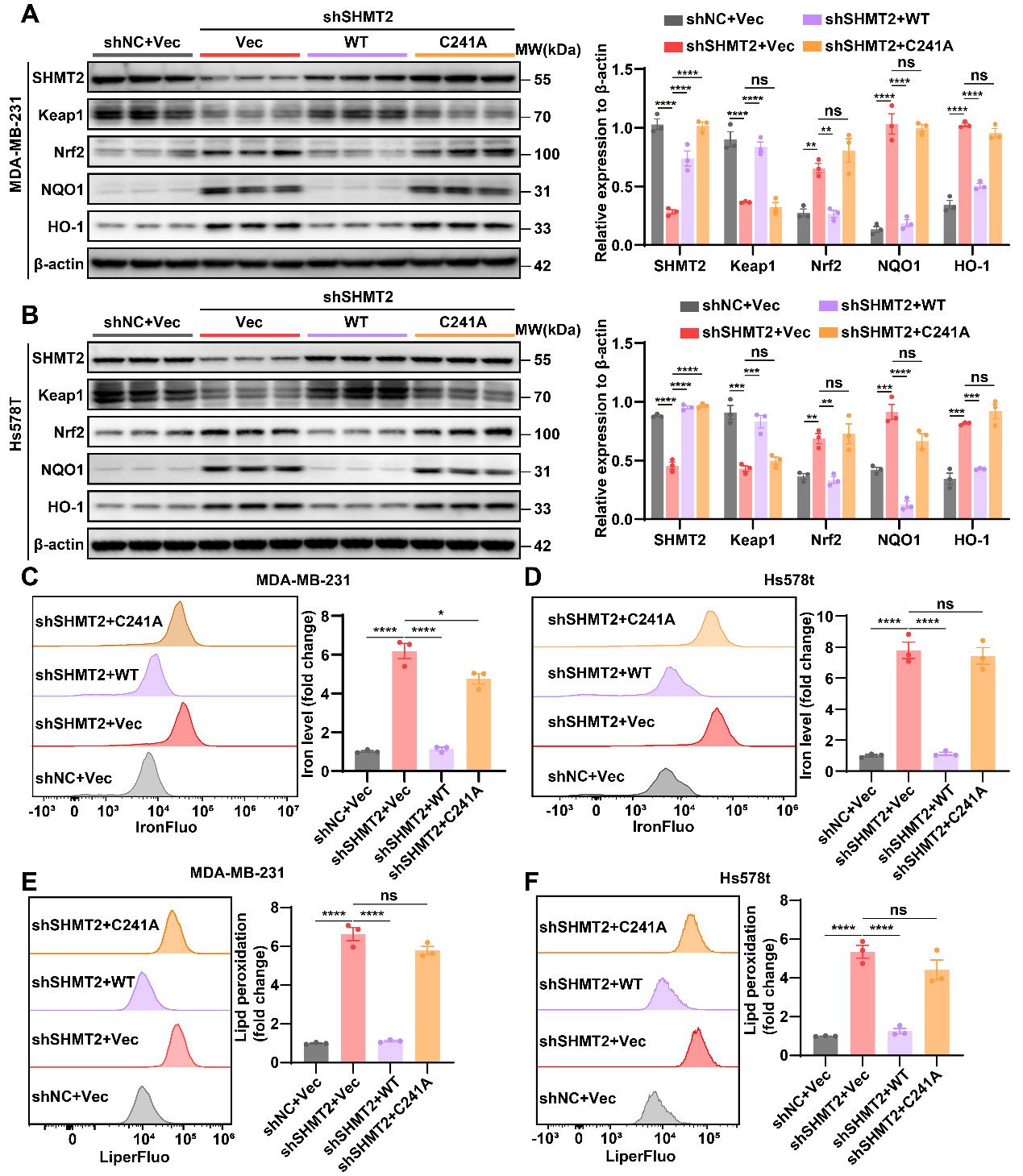


**Fig. S8.** Cysteine 241 is crucial for the functional stability of SHMT2. (A-B) Mutation at cysteine residue 241 significantly activated the Nrf2/HO-1 signaling pathway in TNBC cells. (C-D) Mutation at cysteine residue 241 significantly increased the ferric ion levels in TNBC cells. (E-F) Mutation at cysteine residue 241 significantly induced lipid peroxidation in TNBC cells. Significance levels are indicated as *p < 0.05, **p < 0.01, ***p < 0.001, and ****p < 0.0001.


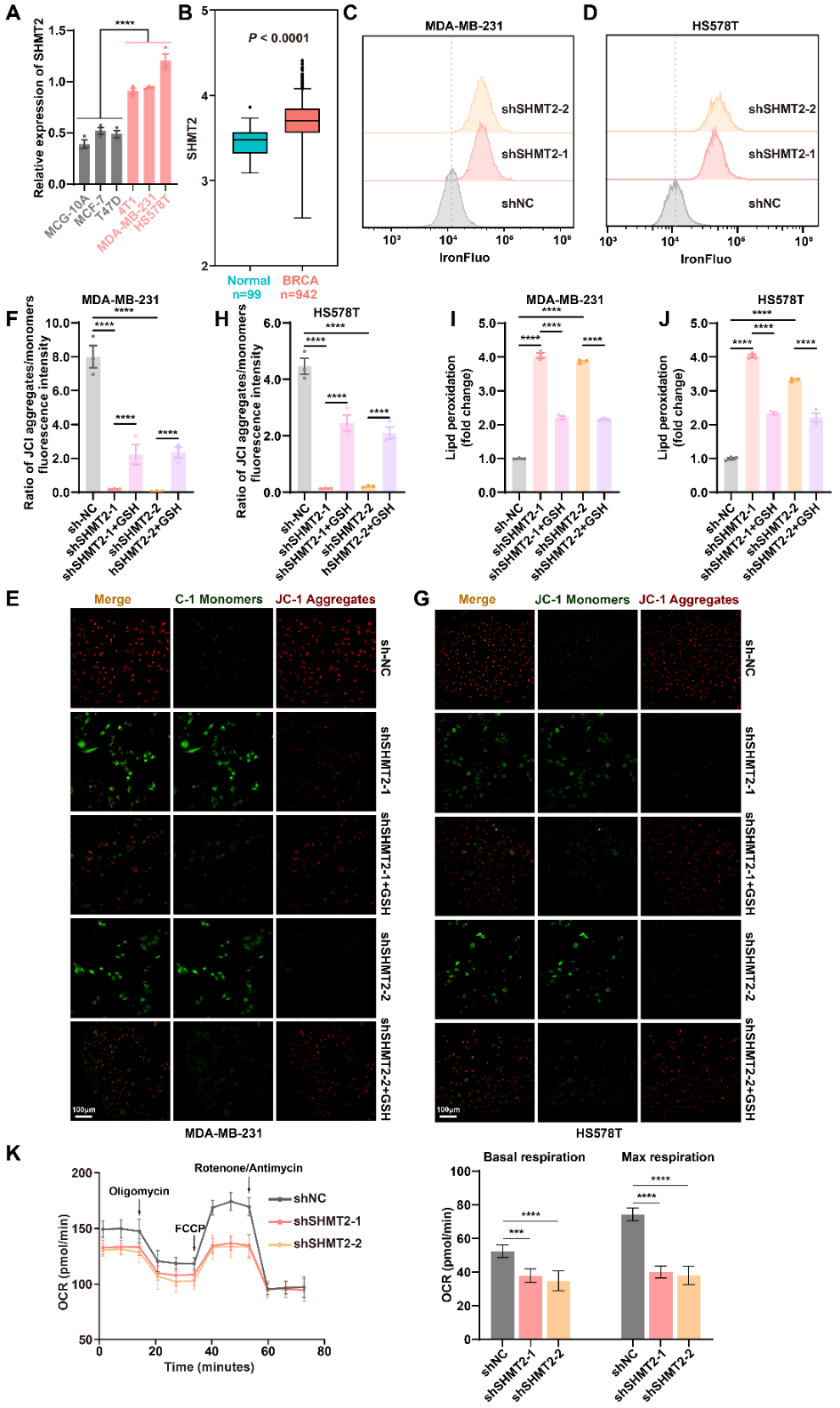


**Fig. S9.** (A) Statistical analysis results for immunoblotting in Fig. 9A. (B) SHMT2 is highly expressed in breast cancer patients. (C-D) Fluorescence intensity distribution map for Fig. 9M. (E-H) Stable knockdown of SHMT2 significantly impairs mitochondrial membrane potential in TNBC cells. (I-J) Statistical analysis results for Fig. 9N-O. (K) The OCR of MDA-MB-231 cells with SHMT2 knockdown. Data were presented as means ± SEM (n = 3). Significance levels are indicated as ^*^p < 0.05, ^**^p < 0.01, ^***^p < 0.001, and ^****^p < 0.0001.


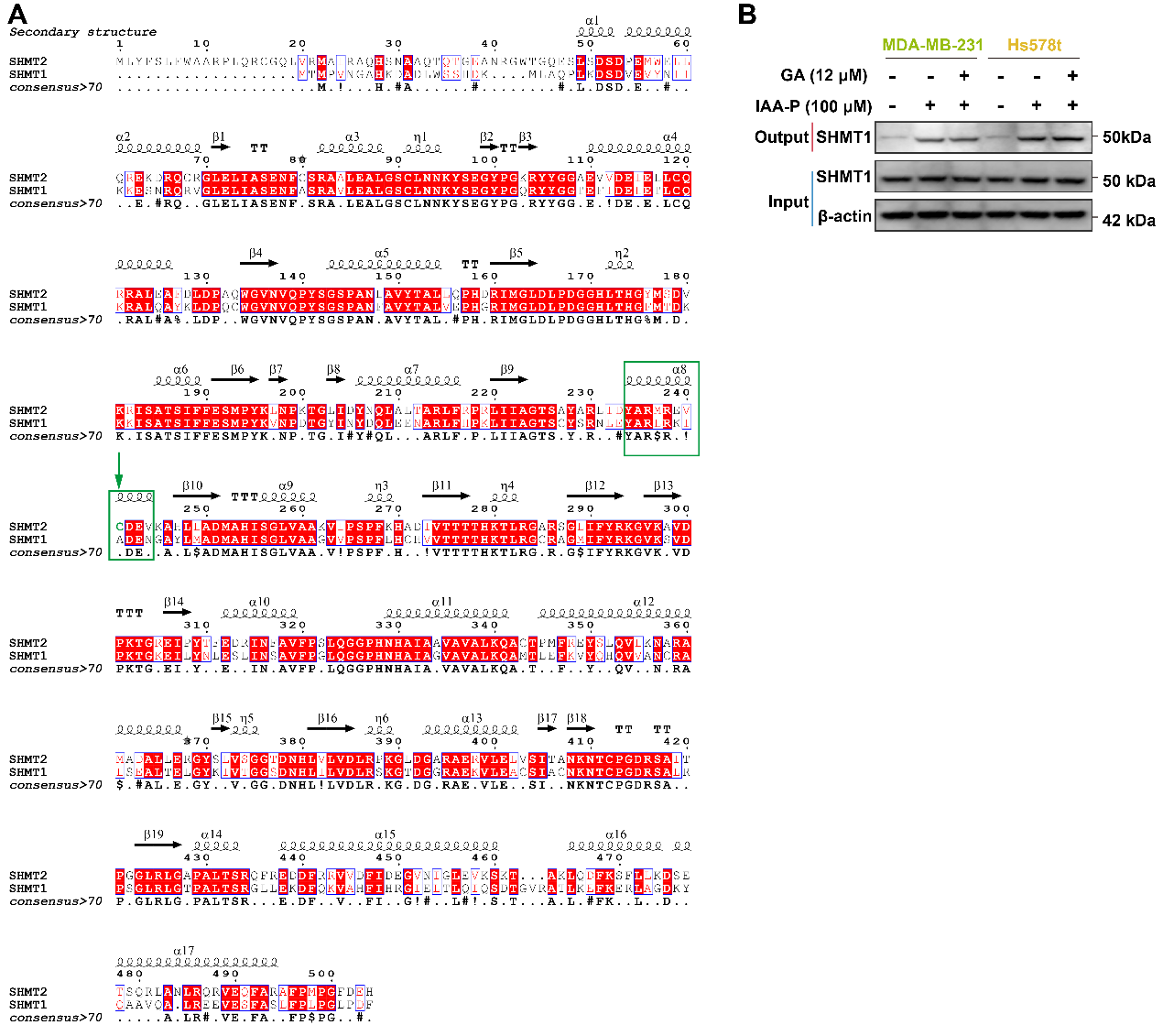


**Fig. S10** GA exhibited specific covalent modification on SHMT2 rather than SHMT1. (A) Protein sequence alignment results for SHMT1 and SHMT2. The green rectangle indicates the eighth α-helix, and the green arrow points to the amino acid residue at position 241. (B) Pull-down-WB confirmed the binding of SHMT1 and GA.


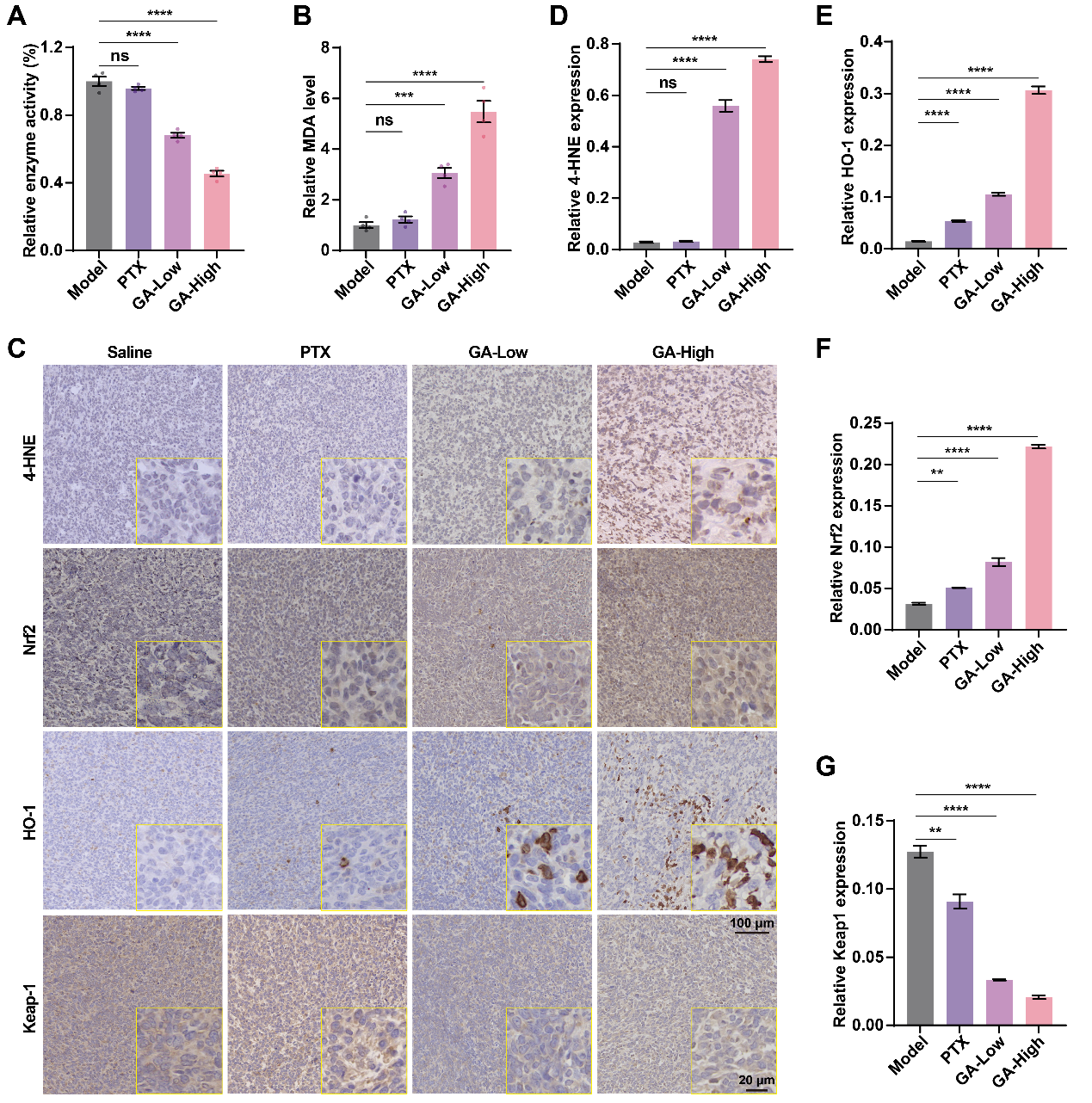


**Fig. S11**. GA inhibits SHMT activity and drives ferroptosis via the Nrf2/HO-1 axis *in vivo*. (A) Analysis of total SHMT enzymatic activity in tumor lysates. (B) Assessment of lipid peroxidation levels in tumor tissues, quantified by relative malondialdehyde (MDA) concentration. (C) Representative IHC staining of tumor sections for 4-hydroxynonenal (4-HNE), Nrf2, HO-1, and Keap1 across treatment groups. (D–G) Quantification of relative IHC staining intensity for 4-HNE (D), HO-1 (E), Nrf2 (F), and Keap1 (G). Data were presented as means ± SEM (n = 3 biologically independent samples). Significance levels are indicated as *p < 0.05, **p < 0.01, ***p < 0.001, and ****p < 0.0001, ns, not significant.


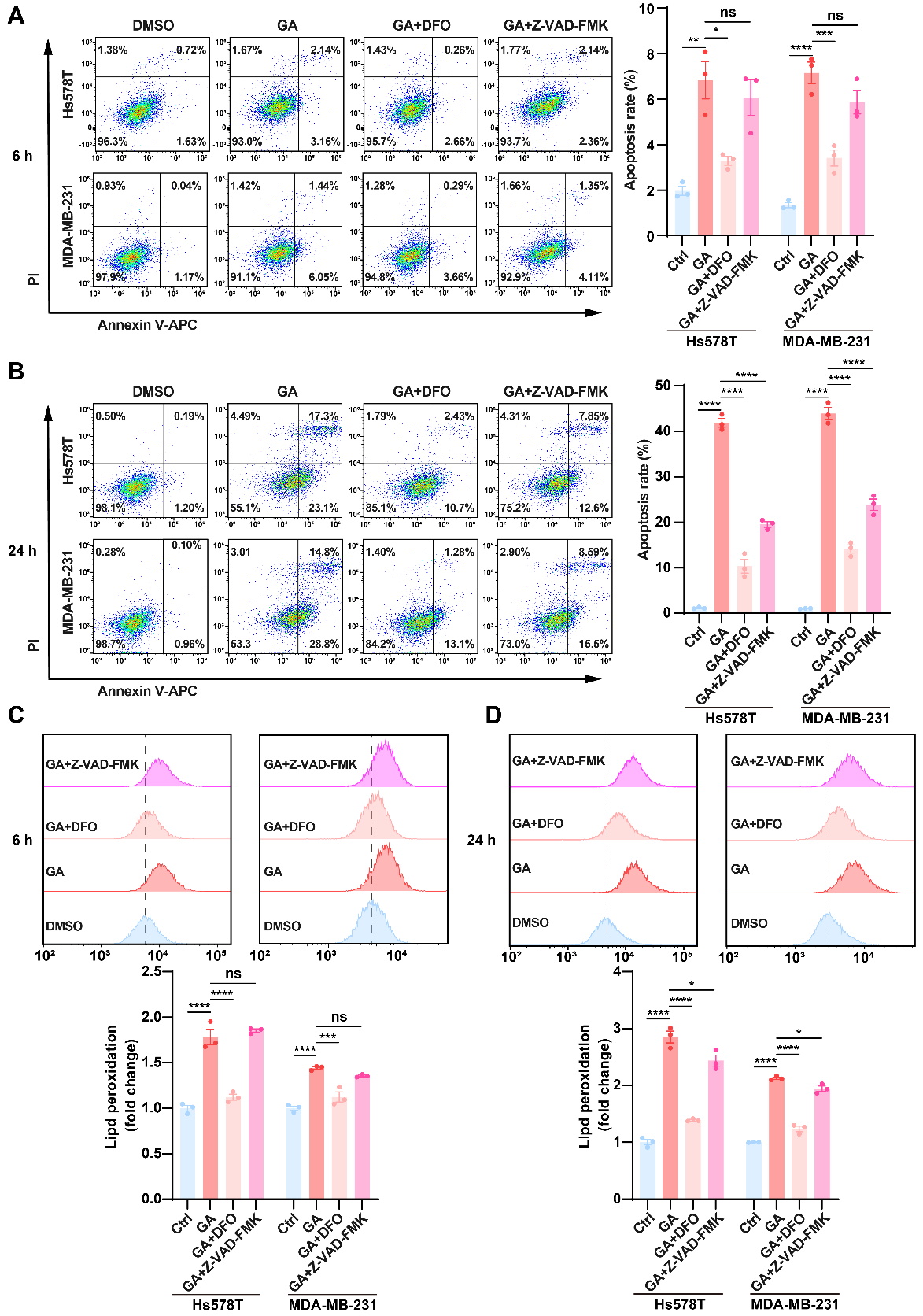


**Fig. S12**. Ferroptosis acts as the primary upstream driver of GA-induced cell death. (A) Flow cytometry analyzed apoptosis in TNBC cells treated with GA for 6 hours under conditions with or without the ferroptosis inhibitor DFO or the pan-caspase inhibitor Z-VAD-FMK. (B) Flow cytometry analyzed apoptosis in TNBC cells treated with GA for 24 hours under conditions with or without the ferroptosis inhibitor DFO or the pan-caspase inhibitor Z-VAD-FMK. (C) Flow cytometric analysis of lipid peroxidation levels in TNBC cells treated with GA for 6 h in the presence or absence of the ferroptosis inhibitor DFO or the pan-caspase inhibitor Z-VAD-FMK. (D) Flow cytometric analysis of lipid peroxidation levels in TNBC cells treated with GA for 6 h in the presence or absence of the ferroptosis inhibitor DFO or the pan-caspase inhibitor Z-VAD-FMK. Data were presented as means ± SEM (n = 3 biologically independent samples). Significance levels are indicated as *p < 0.05, **p < 0.01, ***p < 0.001, and ****p < 0.0001, ns, not significant.
